# Universal patterns of long-distance commuting and social assortativity in cities

**DOI:** 10.1038/s41598-021-00416-1

**Published:** 2021-10-21

**Authors:** Eszter Bokányi, Sándor Juhász, Márton Karsai, Balázs Lengyel

**Affiliations:** 1 Laboratory for Networks, Technology and Innovation, Corvinus University of Budapest, Budapest, 1093 Hungary; 2 Agglomeration and Social Networks Lendület Research Group, ELKH Centre for Economic and Regional Studies, Budapest, 1097 Hungary; 3 Department of Network and Data Science, Central European University, 1100 Vienna, Austria; 4 Rényi Alfréd Institute of Mathematics, Budapest, 1053 Hungary

**Keywords:** Socioeconomic scenarios, Psychology and behaviour

## Abstract

Millions commute to work every day in cities and interact with colleagues, partners, friends, and strangers. Commuting facilitates the mixing of people from distant and diverse neighborhoods, but whether this has an imprint on social inclusion or instead, connections remain assortative is less explored. In this paper, we aim to better understand income sorting in social networks inside cities and investigate how commuting distance conditions the online social ties of Twitter users in the 50 largest metropolitan areas of the United States. An above-median commuting distance in cities is linked to more diverse individual networks, moreover, we find that longer commutes are associated with a nearly uniform, moderate reduction of overall social tie assortativity across all cities. This suggests a universal relation between long-distance commutes and the integration of social networks. Our results inform policy that facilitating access across distant neighborhoods can advance the social inclusion of low-income groups.

## Introduction

Cities are champions of diversity^1–3^. Complex interaction networks of individuals in urban areas enabled by population density, co-location, and easy access together made cities the global engines of technological and economic progress^4–7^. However, cities are also known for high levels of segregation^8–10^ where disparate neighborhoods are separated from each other in the urban space^11–15^. Furthermore, spatial segregation by income also fragments social networks, which can hinder progress and can deepen inequalities^16–20^. Given the importance of this problem, a growing community has investigated the patterns of mobility in cities to better understand mixing potentials across disparate and diverse neighborhoods^21–24^, which may increase economic prosperity^25^. Yet, less is known whether mobility mixing has any imprint on the social connections of people.

Commuting covers a large share of urban mobility^26^ and by connecting home with work locations, the places where people spend most of their time, it plays an important role in the spatial formation of social connections^27–29^. Since aggregated social networks form spatially bounded communities across neighborhoods^17^, the further one commutes, the higher the likelihood that commuting-related social connections will introduce diversity in the egocentric network of the commuter^30,31^. Due to spatial segregation, economically disparate neighborhoods tend to be far from each other^32^, thus long commutes are more likely to link places with different social status^33,34^. Nevertheless, it is not trivial that long commutes should facilitate social inclusion, because social interactions might remain assortative even at places far from home^21,23,35^. Meanwhile, the time to develop new social connections is especially limited for low-income workers who travel to work during rush hours^36,37^.

The spatial distribution of high versus low-income households determines the length of travel that can bridge disparate neighborhoods. Since the scale of socio-economic isolation greatly varies across cities^12^, one may expect that the mobility of people also enables a different degree of social mixture. However, the assortativity of urban mobility is a universal feature across cities: individuals have been recently reported to visit locations that are similar to their home neighborhood^21,23,38–40^. Yet, how assortativity of commuting and social networks are related and how this relation is modified by the length of commute in cities is still largely uncovered.

In this paper, we aim to better understand how mixing in urban social networks is facilitated by commuting. To answer this question, we use a unique dataset on 348,850 Twitter users living in the 50 largest metropolitan areas of the US and track their home and work locations as well as their mutual followership ties on the platform, which from now on, we call the social network of users. We project these social networks in the urban space and attribute users with an average income based on their home locations on an income map extracted from census data. By comparing ego network indicators between people commuting to different distances, we find that long commuting is associated with lower levels of transitivity, the tendency that friends of friends know each other, and higher levels of income diversity among friends. These results are consistent across the 50 largest US cities and suggest that long commutes can indeed facilitate social mixing.

Our results suggest a universal relation between commuting and integration of disparate social networks. The paper contributes to the discussion on the importance of commuting in cities and shows that longer commutes have a measurable even though moderate influence on establishing diverse and less segregated social connections. The findings imply that supporting access to distant work can help the inclusion of lowest income groups and to a certain degree the richest as well, regardless of the urban context.

## Results

We use a unique Twitter database that contains all messages and profile information of 348,850 Twitter users in the top 50 metropolitan areas of the United States. The data was collected between 2012 and 2015 and due to the sample selection method described in^41^, the database contains a considerable amount of individuals who allowed automatic GPS data collection for all their messages. This dataset was used in previous research to detect dominant language use and temporal patterns connected to socio-economic indicators such as ethnicity or unemployment in the US, to establish world-wide communities of users reflecting political and cultural boundaries, and to model the spreading of viral content^14,42–44^.

**Figure 1:**
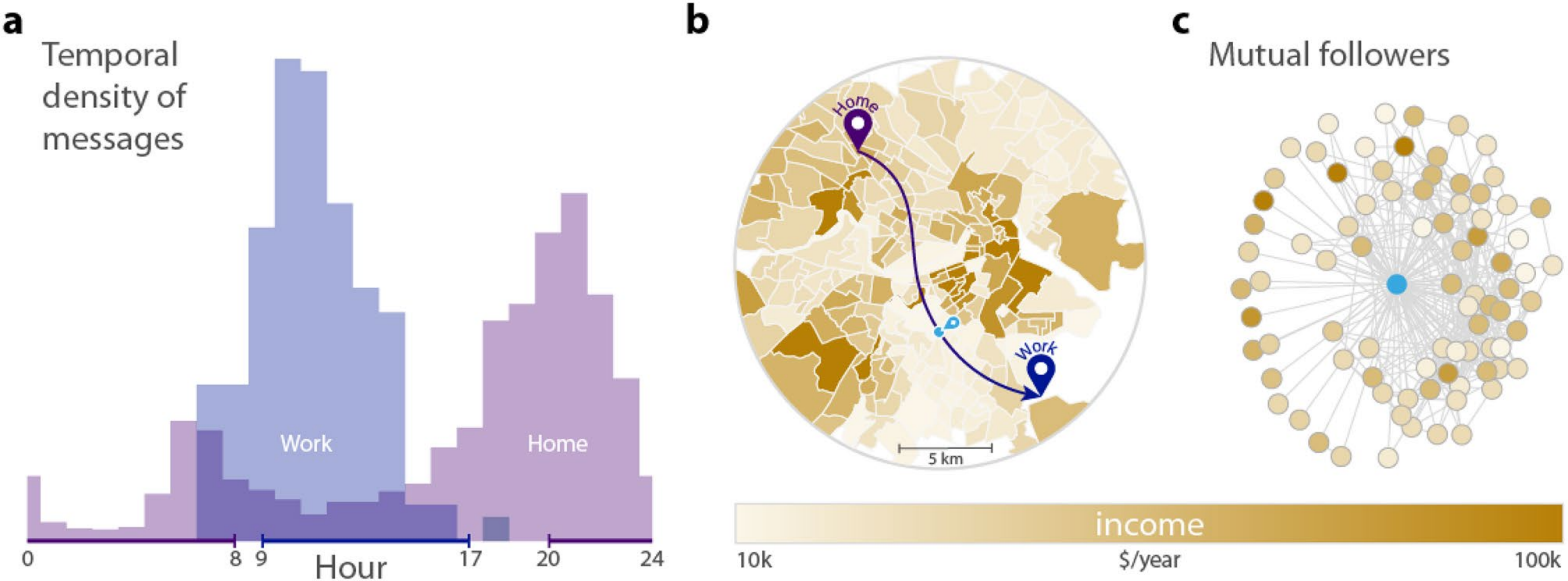
Combination of spatial, temporal and social network data of geolocated Twitter messages. (**a**) Home and work locations of users are identified through the distribution of timestamps on all their collected tweets within their most frequently visited spatial clusters. We assign a possible home location (8 p.m.–8 a.m.) and a possible work location (9 a.m.–5 p.m.) to each user^45,46^ as their most frequently visited location in the given period. The histogram represents the timeline of tweets for the clusters of a sample user. (**b**) Commuting is defined as the overhead distance between users’ home and work locations. The colorbar of the map indicates the income level of census tracts. Census tract shapes have been downloaded from https://www.census.gov/data/developers/data-sets/acs-5year.html, the figure is the authors’ own creation using the geopandas library in Python (geopandas version 0.6.1, https://pypi.org/project/geopandas/0.6.1/, Python 3.7.2). (**c**) Twitter ego network of a sample user based on mutual followership. The coloring of nodes also corresponds to the level of income in the home tract of users.

Figure 1 illustrates how commuting and social network information is retrieved from the data. Home and work locations are detected by the most frequent locations of tweets in the morning and evening hours or during daytime as depicted in Fig. 1a (and as explained in “Materials and methods” section). This process enables us to identify the census tract of home and work locations and attach socio-economic status, measured by the average household income of census tracts from the 2012 American Community Survey. Commuting is characterized by the Euclidean distance between home and work and the socio-economic status of both locations. Finally, we construct the ego network for every user from mutual followership of Twitter profiles and characterize egos and alters by the socio-economic status of their home location. This enables us to quantify social mixing in terms of commuting and social ties in cities.

Figure 1b shows the census tracts of inner Boston colored by the average annual household incomes and the home and work locations of a sample user. The user’s ego network is depicted in panel (c), with colors indicating the income of the neighbors inferred from their home census tract. Each user in our sample has at least 1 mutual followership-based connection and has identifiable home and work locations that are at least 100 m away from each other. The distribution of users across the 50 selected cities is illustrated in Supplementary Information (SI) 1 and 2. For a more detailed description, see “Materials and methods” section.

To characterize the relation between *d*, the distance of commutes, and the social network of individuals, we compare the social networks of people commuting to $d>{median}$ with $d<{median}$ commuting distances in each of the 50 largest US metropolitan areas. Median commuting distances are calculated on the basis of the sampled users in each city as illustrated in SI 3. Our expectation is that commuting may induce more out-of-community independent social ties for commuters, in turn decreasing the transitivity of their egocentric networks. We observe this effect by measuring the local clustering coefficient^47^ for each user, which quantifies the tendency that an individuals’ friends know each other. Another assumption of ours is that these out-of-community ties introduce stronger diversity in ego networks in terms of socioeconomic status of neighbors. We quantify this effect via the average income difference from friends in users’ ego networks, which measures the income similarity of online social connections (for a formal definition see Eq. (1) in “Materials and methods” section).

**Figure 2:**
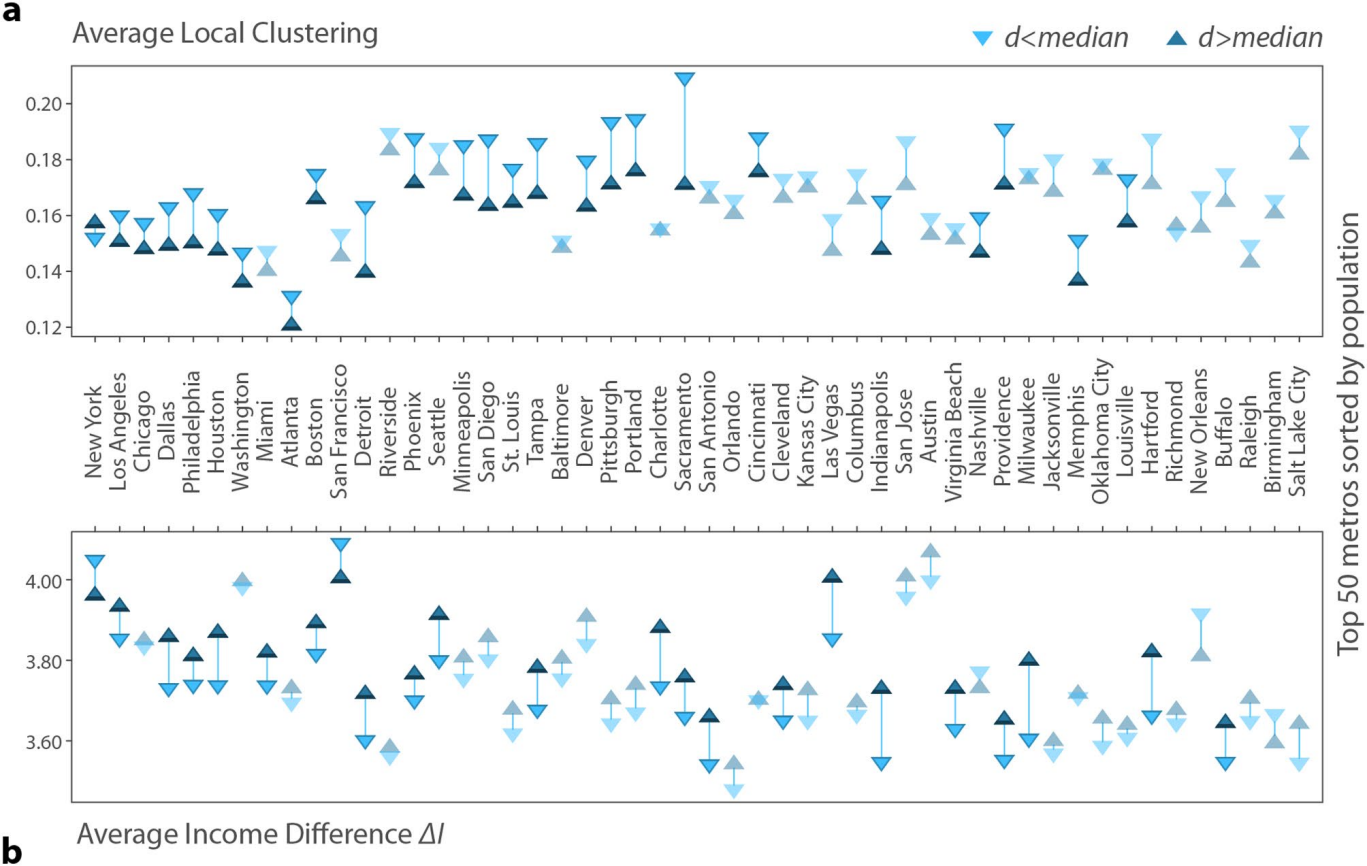
Network characteristics of users and commuting distance in the top 50 metropolitan areas of the United States. (**a**) Network closure measured by the local clustering coefficient is lower in most cities for those users who commute further than the local median distance. (**b**) Income mixture, measured by average income difference from friends, is higher of those who commute above the local median distance in the majority of metropolitan areas. Non-transparent symbols indicate that t-tests suggest significantly different means for the groups $(p<0.05)$.

Figure 2a reports the average of local clustering coefficient and (b) the average income differences of users commuting above and below the local median distance in the 50 largest metropolitan areas in the USA. These findings suggest that, with a few exceptions, an above-median distance commute is associated with lower local clustering (Fig. 2a), and with greater income difference in the commuters’ ego networks (Fig. 2b). This implies that working further away from home helps people to develop less cohesive and income-wise more diverse social networks in most metro areas. Note that here metropolitan areas are sorted in decreasing population order and non-transparent markers denote significant differences (p $<0.05$) between averages.

While these results suggest clear trends, they also highlight the heterogeneity of cities. To support these observations, in SI 4, we compute the degrees for below and above median distance commuters, illustrate the underlying distributions, and we also repeat the measurements and find them to be robust for various distance thresholds. A multivariate regression analysis using continuous variables in SI 5 provides further evidence that commuting distance correlates negatively with local clustering even when controlling for the number of connections and income. These regressions also inform us that commuting distance facilitates mixing in social networks by enabling commuters to make more friendships.

For a more detailed insight into the structure of social and mobility assortativity in these cities, next, we analyze social mixing through commuting and online social ties between income groups. We sort all census tracts into income deciles based on the income distribution across all census tracts in the metro area in question and assign an income decile ranging from 1 to 10 to home and work locations. For each metro area, we construct a commuting assortativity matrix *C* and a social network assortativity matrix *S* to represent connection probabilities between these income deciles. The elements of the commuting assortativity matrix $C_{ij}$ measure the probability that a user with home census tract in income decile *i* commutes to work in a census tract of income decile *j*. Similarly, elements of the social network assortativity matrix $S_{ij}$ represent the average probability that a person living in a tract with income decile *i* has a mutual followership tie with a user living in a tract with income decile *j*. For more details on the construction of the matrices, see “Materials and methods” section.

**Figure 3:**
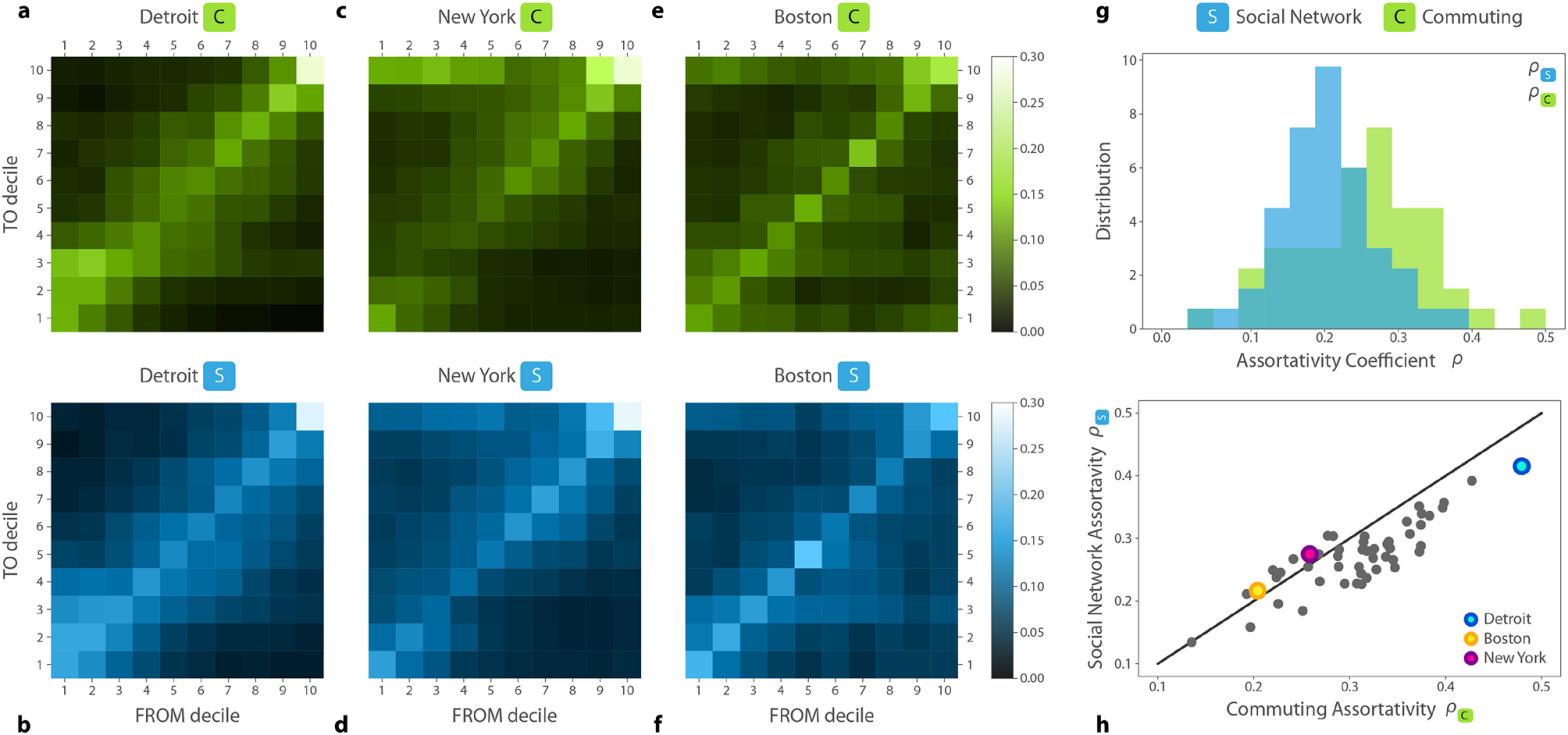
(**a**) Commuting assortativity matrix *C* and (**b**) social network assortativity matrix *S* between the 10 income deciles for Detroit, New York (**c**,**d**) and Boston (**e**,**f**). (**h**) Distribution of Pearson correlations $\rho _C$ (green) and $\rho _S$ (blue) for the assortativity matrices *C* and *S* of the top 50 metropolitan areas of the US. (**g**) Commuting assortativity and social network assortativity are strongly correlated across cities. Solid line represent $\rho _C=\rho _S$.

The aggregated patterns of commuting *C* and friendship ties *S* are presented in Figures 3a-f for three example metropolitan areas, Detroit, New York, and Boston. Unlike previous studies^23,35^, we do not observe universal assortativity patterns over all cities in these networks. In some of the cities, such as Detroit, the strong diagonal component features strong segregation patterns, meaning that people tend to commute to neighborhoods with similar annual household incomes as their home neighborhood, and they tend to form social ties with people living in neighborhoods with similar income, as also found in^24^. In cities like Boston, patterns of mobility and online social ties are less assortative with higher likelihood for diverse, off-diagonal connections. All commuting and social network matrices are available in the SI 6 for the 50 metropolitan areas.

To explore this heterogeneity further, we computed the Pearson correlation coefficient of the above matrices (see “Materials and methods” section Eq. (4)). We use these correlation coefficients as a single-number measure of assortativity in the metropolitan-level networks denoted by $\rho _C$ for the commuting, and $\rho _S$ for the social network assortativity matrix. We show the $\rho _C$ and $\rho _S$ distributions in Fig. 3g. We see here that the level of assortativity varies remarkably across the 50 metro areas, but judging by their averages, commuting in metro areas (${\bar{\rho }}_C=0.31\pm 0.07$) are more income assortative than online social ties (${\bar{\rho }}_S=0.27\pm 0.05$). Interestingly, our observations in Fig. 3a–f further suggest that the measured commuting and social network assortativity matrices are not independent from each other. Indeed, Fig. 3h illustrates that $\rho _C$ and $\rho _S$ pairs are strongly correlated ($\rho$=0.84) suggesting a substantial relationship that social networks are segregated in cities where home-work commuting patterns are assortative.

**Figure 4:**
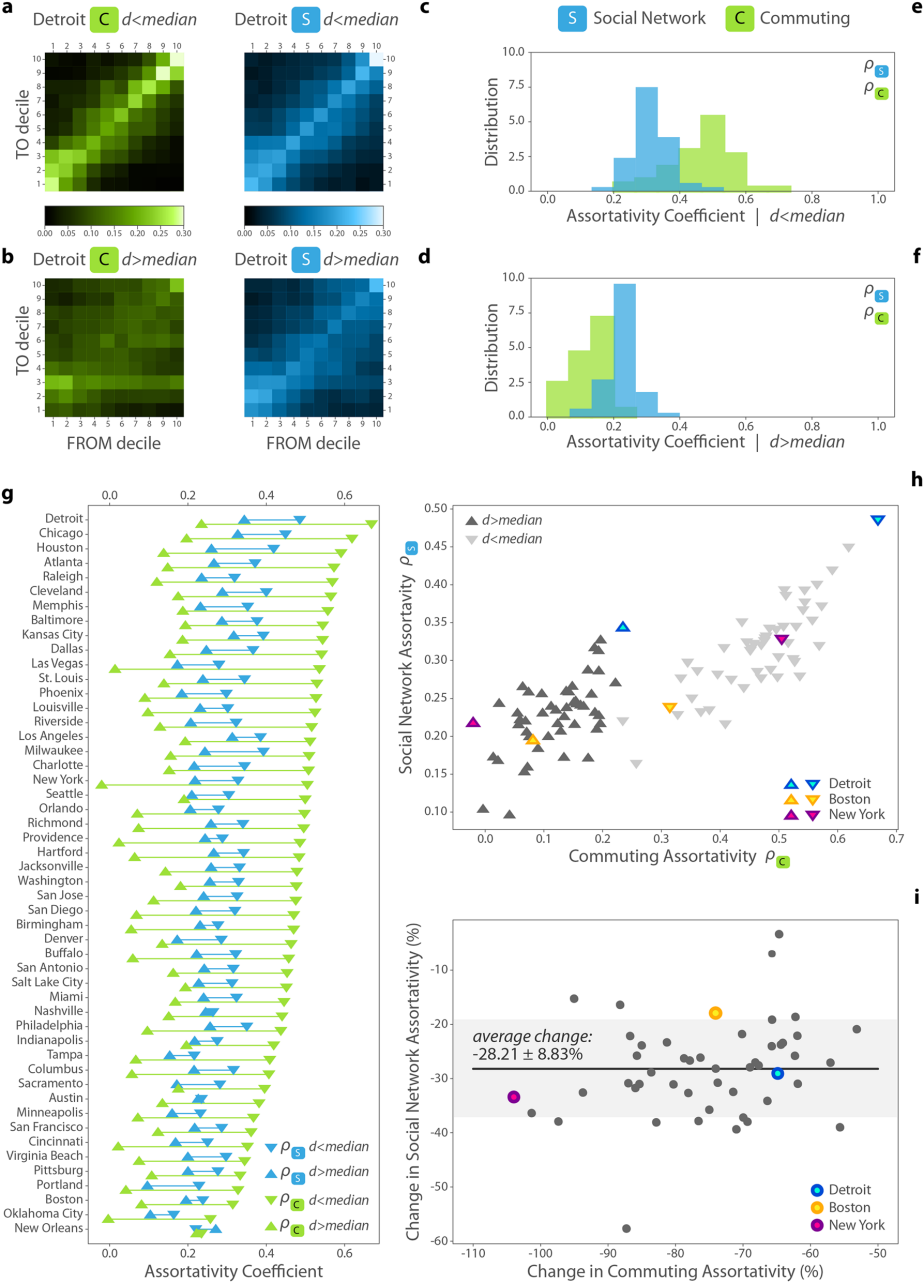
Panels (**a**–**d**) show the *C* and *S* assortativity matrices for the below ($d<median$) and above median ($d>median$) commuting users in a selected metropolitan area, Detroit. (**e**,**f**) The corresponding distributions of $\rho _C$ (green) and $\rho _S$ (blue) for all 50 metropolitan areas for users with $d>{median}$ and $d<{median}$. (**g**) Pairwise values of $\rho _C$ and $\rho _S$ for users with $d>{median}$ and $d<{median}$ by metropolitan areas. Metropolitan areas are sorted in decreasing order by $\rho _C$ for easier representation. (**h**) Social network assortativity versus commuting assortativity for below and above median commuters with selected cities from Fig. 3 labeled. (**i**) Decrease in the commuting assortativity and the social network assortativity measured in percentage. Black horizontal line corresponds to the average change in social network assortativity. Grey shaded area marks the standard deviation.

To investigate the association between long-distance commute and social mixing on the aggregate city-level in more detail, we separate the baseline sample of the *C* and *S* matrices by commuting distance. Thus, we create a *C* and *S* matrix from users commuting to a distance $d<{median}$ and $d>{median}$, as in the example in Fig. 4a–d, where we show these four matrices (two for both *C* and *S*) for Detroit. These matrices indicate that for users commuting an above median distance, matrices are less diagonal, and reflect more diverse and less segregated commuting and social connections. Panels (e) and (f) from Fig. 4 present the distributions of $\rho _C$ and $\rho _S$ for the two subgroups of users in all 50 metropolitan areas. As expected, longer commuting distance is associated with less assortativity because distant workplaces are likely to be located in socio-economically different environments as compared to home location. This might be due to spatial clustering of tracts with similar annual household incomes^12^, leading to shorter commute patterns landing in places with similar income level. In parallel, we observe that longer commutes are also associated with lower levels of assortativity of online social network ties such that off-diagonal social ties are relatively more likely for $d>{median}$ distances than for $d<{median}$. However, while $\rho _C$ falls sharply for $d>{median}$ distances compared to $d<{median}$, the difference of $\rho _S$ is moderate in Fig. 4e,f. This finding indicates that although long-distance commutes can link disparate neighborhoods, not all of the diversity generated by commuting has imprints on social connections. Instead, income homophily remains a major yet weaker factor of social tie selection for long commuters as well.

Despite the heterogeneity of metro areas, results in Fig. 4g show general patterns in two regards. First, assortativity of both commuting and social networks are lower for long-distance commuters in every metropolitan area. Second, the assortativity reduction between shorter and longer than median commutes is decreasing sharply, while the reduction of social network assortativity is moderate and takes similar values for every metropolitan area. The Pearson correlation coefficient between the two assortativity values $\rho _S$ and $\rho _C$ is 0.80 for short commuters and 0.72 for long commuters, thus they signify a strong relationship between mobility and social network assortativity patterns for both user groups (Fig. 4h). To understand the magnitudes of change, we calculate the percentage of social network assortativity reduction by ($(\rho _{S,d>median}-\rho _{S,d<median}) / \rho _{S,d<median}$) and the percentage of commuting assortativity reduction by ($(\rho _{C,d>median}-\rho _{C,d<median}) / \rho _{C,d<median}$) for each city. Illustrating these metrics, Fig. 4i shows that the decrease in commuting assortativity ranges on a wide scale between − 50 and − 100%. However, the decrease in the social network assortativity concentrates around the average value of $-28\pm 9\%$. Remarkably, this signals a universal pattern of social mixing potentials across very different urban settings and it explains a general trend of how mixing through commutes manifests in social inclusion. SI 7 illustrates that the uniform $\sim 30\%$ decrease disappears if we separate two user groups randomly instead of by commuting distance, but this observation remains consistent across multiple absolute distance thresholds (3 km, 5 km, and 10 km). In addition, in SI 8, we show that assortativity reduction by long-distance commute is a result of increasing social mixing of users from poorest and to some extent, from the richest neighborhoods.

## Discussion

Understanding the complex behavioral patterns of people is crucial to develop more liveable, equal and sustainable urban environments. Our study contributes to this challenge by using large-scale geolocated Twitter data to study the role of commuting in the composition and assortativity of social interaction. We illustrate that long-distance commuting acts against structural closure and income homophily of social relationships and reduces segregation between remote income classes by facilitating connections and mixing. We show that home-work commutes and online social ties are not equally assortative in every metropolitan area, but in most cases, commuting is even more likely to point to places with similar income level than online social connections. Our findings suggest that longer commutes are more likely to connect places with different income levels, which contributes to the development of more diverse and less assortative social ties. Moreover, working further away from home results in more heterogeneous social connections in every metropolitan area.

Our results suggest that urban mobility has a fundamental role in fulfilling the promise of social inclusion and reduction of social segregation in cities. The association between commuting distance and social networks is remarkably stable across all metropolitan areas with different size and spatial structure^48^. This universal pattern highlights that commuting-enabled social mixing follows similar mechanisms regardless of the urban context. We find that facilitating the access between distant neighborhoods can reduce segregation in metropolitan areas, while gains in social inclusion are limited to a 30% reduction of assortativity. These results signal that providing access across disparate neighborhoods cannot erase mechanisms of social network segregation but can mitigate the divide between rich and poor.

The methodology applied in this paper could easily be extended to other cities with large populations of geolocated Twitter users, and where granular census data with similar spatial resolution is available. However, this approach is not without limitations. While we are confident in our approach to identify home and work locations of users, we cannot confirm whether the identified work locations are actual workplaces or any other facility that people visit frequently during daytime (such as restaurants, schools, etc.). We measure commuting distances as the Euclidean distance between the home location and the work location, whereas in multiple cities, physical obstacles such as rivers might considerably increase travel times or change the socio-economic segregation patterns of settlements^49^. We are not aware of the available modalities to reach work destinations, but we admit that it would also introduce a large variability into travel times. We choose this simplification because both travel times with a car or public transportation might depend on the exact time of the day and varying traffic conditions. Both the underestimation of commuting distances and the inclusion of users who might not have a regular workplace can result that the observed commuting in our case (see SI Figure 3) falls behind the commuting distances reported in the American Community Survey.

Because we do not use an absolute threshold to distinguish long and short commutes, and we use the city-wise median to divide the users into categories, we believe that the aforementioned biases do not affect our results drastically. However, we test both the results of Figs. 2 and 4i for different absolute distance thresholds, 3 km, 5 km and 10 km, where our results still hold (see SI 4 and SI 7).

Even though the fraction of users present in the analysis is proportional to the population size of the 50 metropolitan areas (see SI Figure 2), we have to highlight that our dataset is not fully representative for the US population and results have to be interpreted accordingly. Hargittai and Litt^50^ finds that African American users are overrepresented on the platform, and Twitter users are predominantly young, well-educated^51,52^ and unrepresentative of other ethnicities^53,54^. Therefore, we cannot generalize our findings to the whole population of these metropolitan areas. Another limitation of the study could be that the free 1% sample from Twitter Streaming API was used for the initial data collection. Joseph et al. and Morstatter et al.^55,56^ confirms that tweets filtered to containing GPS coordinates are retrieved to almost 90% of the time compared to the full dataset. By imposing strict count limits, spatio-temporal constraints and mutual followership for ties, we believe that our sample is less distorted from bot activity than what^57^ would suggest.

Despite the imperfection of the data, we believe that the presented exercise offers useful insights to the structure of social connections within urban areas. Such large-scale, micro-level analysis enables us to uncover the fundamental patterns behind segregation, inequality or the lack of inclusion inside cities. Publicly available online social network data can complementing official census reports or surveys and can provide opportunities to detect and react to societal patterns and changes.

## Materials and methods

### Data collection and combination methods

We focus on users of the online social networking site of Twitter who posted tweets frequently containing precise geographic information. More specifically, we use a unique, historical database rich in tweets containing GPS coordinates^41,58^. These tweets originate from users who enabled the exact geolocation option on their smartphones. Overall, we detect the three most frequent tweeting locations of users as spatial clusters of their locations in the 50 most populated metropolitan areas of the United States. We use the Friend-of-Friend algorithm^59^ to cluster the spatial coordinates for each user. This algorithm is a paralellizable, scalable clustering algorithm known from astronomy, and it is widely used to identify galaxy clusters^60^. In our case, any two tweet coordinates of the same person are considered to belong to the same spatial cluster if their separation is less than 1 km. For each cluster, we determine the first two moments of the coordinate distribution. Before calculating the mean coordinates of the cluster, we trim data points until all points are inside a $3\sigma$ radius to eliminate outliers. We keep the aforementioned three highest cardinality clusters per user^41,42^.

To determine the possible home and work locations of users, we follow the approach proposed by^46^. We assume that the home and work locations of users are within the previously detected three clusters. We select users for whom at least two out of the three clusters are within the same metropolitan area from the top 50 metropolitan areas of the United States and one of these clusters is their top cardinality location. First, we calculate the daily timeline of clusters for each user based on the timestamp of the tweets with hourly aggregation, converting all UTC tweet timestamps to local times across the whole US. We only consider users with more than 15 tweets on weekdays (Monday to Friday) in total. Local aggregated weekday timelines of two clusters for a sample user are presented in Fig. 1a. We calculate the share of tweets sent between 9 a.m. and 5 p.m. on weekdays to capture messages predominantly sent during the working hours. Similarly, we calculate the share of tweets sent between 8 p.m. and 8 a.m. on weekdays contributing to a possible home tweeting fraction. Then, the cluster with the highest work tweet share or home tweet share becomes the work and home cluster of the user.

Commuting of users is characterized by the overhead distance between their home and work locations. We restricted our sample to users with at least 0.1 km commutes to avoid those ambiguous cases where detected home and work clusters are the same. Thus, we have 975,492 users in our sample. The distribution of observed commuting distances for each metro area are presented in SI 3. Additionally, we attach socio-economic data to each home and work location in the observed metropolitan areas from the 2012 American Community Survey. More precisely, we map the home locations of users into the census tracts of the top 50 US metropolitan areas and attribute the average annual household income of the census tract to each user living there. After that, we sort users into city-wise income deciles based on the average annual household incomes, and we apply the same approach to determine the average income and the income decile of their workplaces. Figure 1b shows the commute of the same sample user and the income level of the surrounding census tracts.

Social connections of users are defined as their mutual followership relations on Twitter as they represent relative stronger ties in context of online social networks^61^. Figure 1c represents a sample ego network that we construct for every user from our home-work sample who has at least 1 mutual followership tie within the same metropolitan area. In the end, we have 348,850 users for whom we have both the home and work location information, and a mutual followership ego network. The composition and spatial distribution of our final sample is presented in SI 1. Through the home location of the user’s friends, we can infer their income, thus, we are able to characterize the socio-economic status of the neighbors in the ego networks by identifying their income deciles. Figure 1c shows this characterization by using the same colorscale for both the ego and its first neighbors as the choropleth map in Fig. 1b.

At the individual level, commuting and online social ties of our users are characterized by multiple different indicators. We measure user commutes by the Euclidean distance *d* between their inferred home and work locations. We calculate degree and local clustering coefficient from their ego networks. We also measure the average income difference between their own home income and the home income of their friends, following the formula below:

1 $$\begin{aligned} \Delta I =\frac{1}{\#\text {neighbors}}\sum _{f\in \text {neighbors}} \log _{10}\left| I_f-I_{ego}\right| \end{aligned}$$

### Assortativity metrics

At the aggregated, metropolitan area level, we create multiple different assortativity matrices between income deciles *D* for each metropolitan area. First, an assortativity matrix of commuting is constructed, where we capture the probability $C_{ij}$ that a user *u* belonging to a home census tract in income decile $D=i$ commutes to a tract with income decile $D=j$ to work. Second, we measure the conditional probabilities of social ties across home census tracts in different income deciles, the social network assortativity matrix *S*. The element $S_{ij}$ of this matrix measures the probability that a user *u* from income decile $D=i$ has a mutual followership tie to a user in income decile $D=j$. Formally, the two matrices can be calculated as

2 $$\begin{aligned}  C_{ij}&= \frac{ \sum \nolimits _{ \left\{ u \in U\left| D_{u,\mathrm {home}=j}, D_{u,\mathrm {work}=i} \right. \right\} } 1 }{ \sum \nolimits _{ \left\{ u \in U \left| D_{u,\mathrm {home}=j} \right. \right\} } 1 }\end{aligned}$$

3 $$\begin{aligned} S_{ij}&= \frac{ \sum \nolimits _{ \left\{ u \in U \left| D_{u,\mathrm {home}}=j\right. \right\} } \frac{1}{k_u} \sum \nolimits _{ \left\{ e_{uf} \in E_u \left| D_{f,\mathrm {home}=i} \right. \right\} } 1 }{ \sum \nolimits _{ \left\{ u \in U\left| D_{u,\mathrm {home}=j} \right. \right\} } 1 }, \end{aligned}$$

where *U* is the user set within a metropolitan area for which we calculate the matrices, $E_u$ is the set of edges connected to the user *u*, $k_u$ is the degree of ego user *u* in the ego network, $e_uf$ is the undirected edge between user *u* and *f*, $D_u$ and $D_f$ are the (home or work) deciles of users *u* and *f*, respectively. We also measure two additional friendship and commuting assortativity matrices, $S^{d>\mathrm {median}}$, $S^{d<\mathrm {median}}$, $C^{d>\mathrm {median}}$ and $C^{d<\mathrm {median}}$, for users commuting more or less than the median commute in the given metropolitan area. In these cases, the set *U* is what is different in the matrices from Eq. (3).

We measure assortativity in these matrices by calculating the Pearson correlation coefficient $\rho$ of the matrix entries. If we normalize the elements of matrix *X* such that ${\tilde{X}}_{ij} = X_{ij}/n$, where $n=\sum _{i,j}X_{ij}$, the sum of the elements of a matrix, then $\rho$ captures how diagonal these matrices are:

4 $$\begin{aligned} { \rho _X = \frac{\sum \nolimits _{i,j}ij {\tilde{X}}_{ij}- \sum \nolimits _{i,j}i {\tilde{X}}_{ij}\sum \nolimits _{i,j}j {\tilde{X}}_{ij}}{\sqrt{\sum \nolimits _{i,j}i^2 {\tilde{X}}_{ij}- \left( \sum \nolimits _{i,j}i {\tilde{X}}_{ij}\right) ^2}\sqrt{\sum \nolimits _{i,j}j^2 {\tilde{X}}_{ij}- \left( \sum \nolimits _{i,j}j {\tilde{X}}_{ij}\right) ^2}}}, \end{aligned}$$

where the summation for *i* and *j* both go over all of the income deciles $D=1,\dots ,10$. An assortativity value $\rho =+1$ would mean a completely diagonal, thus, completely assortative matrix, whereas $\rho \approx 0$ values indicate the lack of any preference for people following others from the very same income class of their own.

## Supplementary Information

Supplementary Information.
