# Supplementary material for: Universal patterns of long-distance commuting and social assortativity in cities
Source: Sci Rep. 2021 Oct 21;11:20829. doi: 10.1038/s41598-021-00416-1 (PMC8531448; doi:10.1038/s41598-021-00416-1)
Supplement: Supplementary file 1 — Supplementary Information. [file 41598_2021_416_MOESM1_ESM.pdf]

# Supplementary Information
# Universal patterns of long-distance commuting and social assortativity in cities

Eszter Bokányi[1,2,*], Sándor Juhász[1,2], Márton Karsai[3,4], Balázs Lengyel[1,2]

[1]Corvinus University of Budapest; Laboratory for Networks, Technology and Innovation
Budapest, H-1093, Hungary
[2]ELKH Centre for Economic and Regional Studies, Agglomeration and Social Networks Lendület Research Group
Budapest, H-1097, Hungary
[3]Central European University; Department of Network and Data Science
Vienna, A-1100, Austria
[4]Rényi Alfréd Institute of Mathematics,
Budapest, H-1053, Hungary
[*]Corresponding author: bokanyi.eszter@krtk.hu

# Supplementary information

## SI 1: Observed users across the top 50 US metropolitan areas

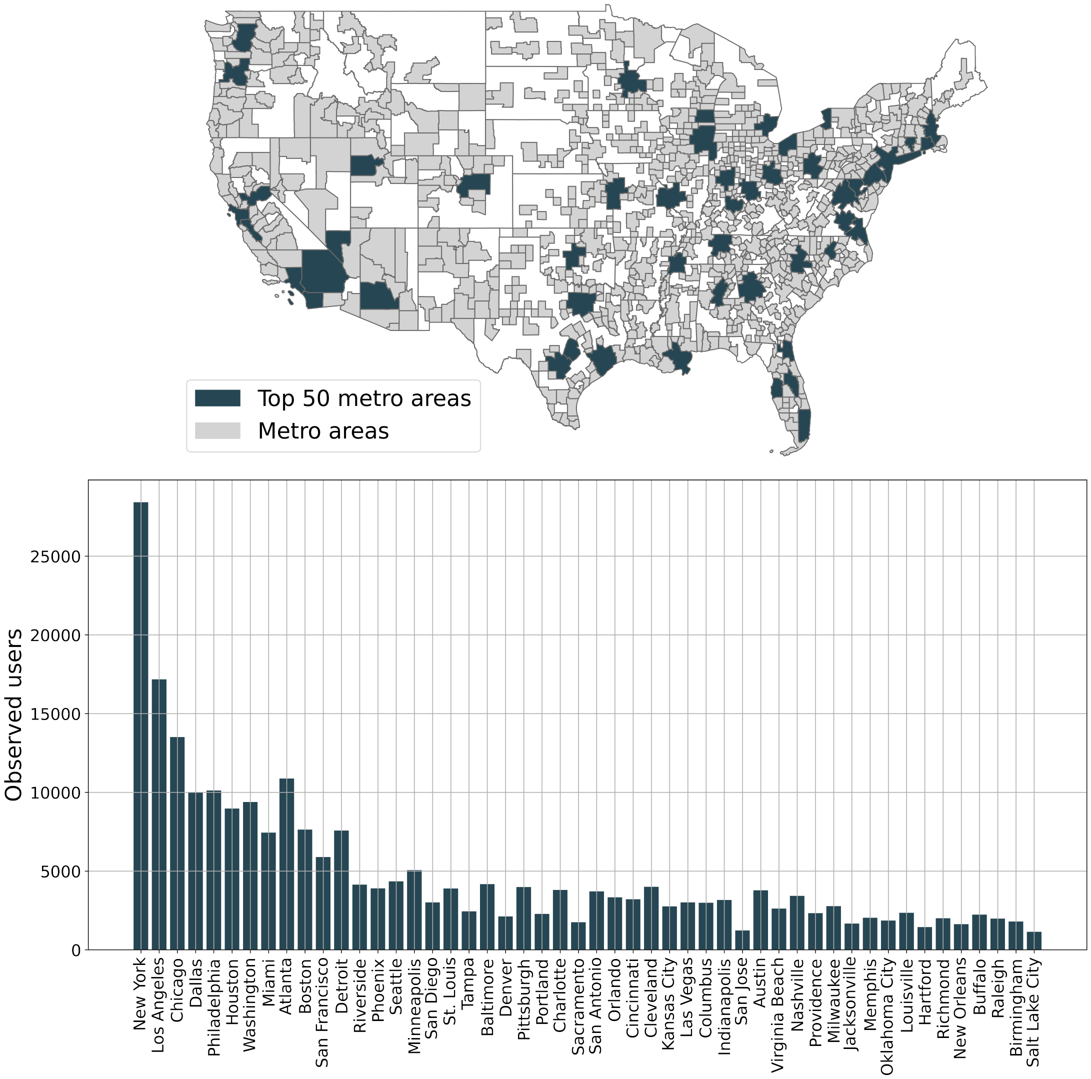


Figure 5: (A) Map of the selected 50 metropolitan areas with the highest population in the US. The map shapefile has been downloaded from https://catalog.data.gov/dataset/tiger-line-shapefile-2019-nation-u-s-current-metropolitan-statistical-area-micropolitan-statist, the figure is the authors' own creation using the `geopandas` library in Python (`geopandas` version 0.6.1, https://pypi.org/project/geopandas/0.6.1/, Python 3.7.2). (B) The histogram represents the number of observed users with home and work locations, minimum 100 meter commute and minimum 1 connection to a user with discovered home and work locations in the same metro area. The metro areas are ordered by population.

## SI 2: Population and observed users in metro areas

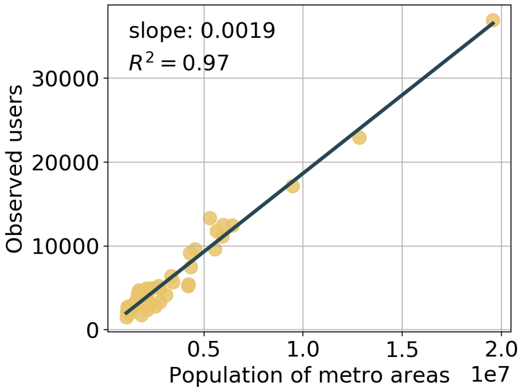


Figure 6: Population size and observed users in the selected 50 metropolitan areas of the US. Observed users have detected home and work locations, commute at least 100 meter and have at least 1 friendship tie to users with discovered home and work locations inside the same metro area.

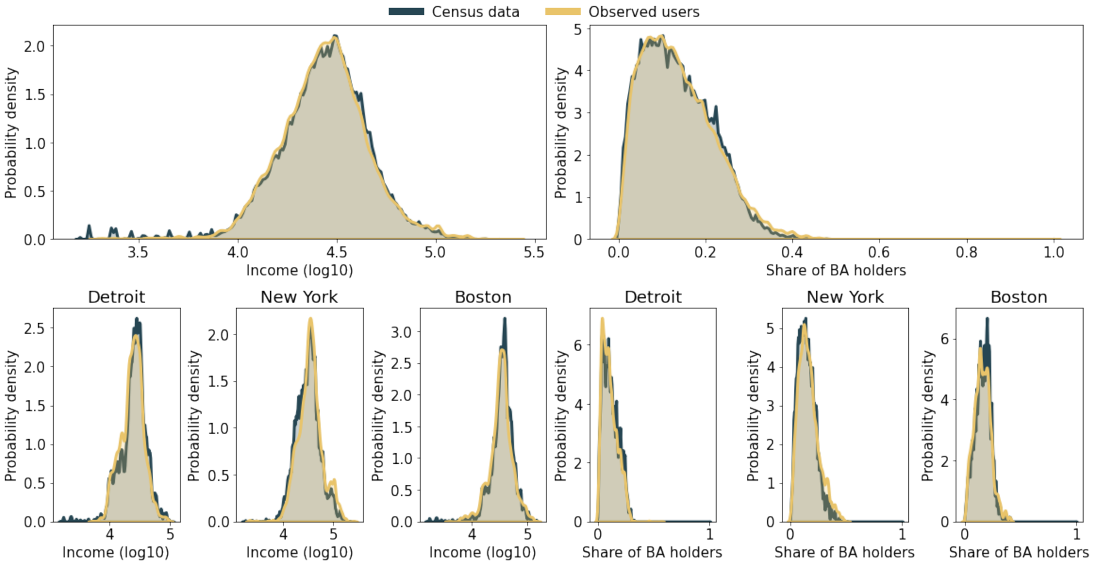


Figure 7: Comparison of the distribution of income and education statistics between official census data and our observed users in the 50 metropolitan areas of the US. The first row presents the population weighted income distribution of census tracts and the population weighted share of BA educated people in census tracts across all of the 50 metropolitan areas. The second row illustrates the same statistics for the three example metro areas of Detroit, New York and Boston. Kolmogorov–Smirnov tests suggest that the distributions are not identical, but the visualizations show that they are very close. This suggests that the analyzed data on Twitter users do follow the general distributions of the urban population but low-income groups are slightly under-represented while highly educated groups are slightly over-represented. However, this pattern is not systematic across cities.

## SI 3: Distribution of commuting distances

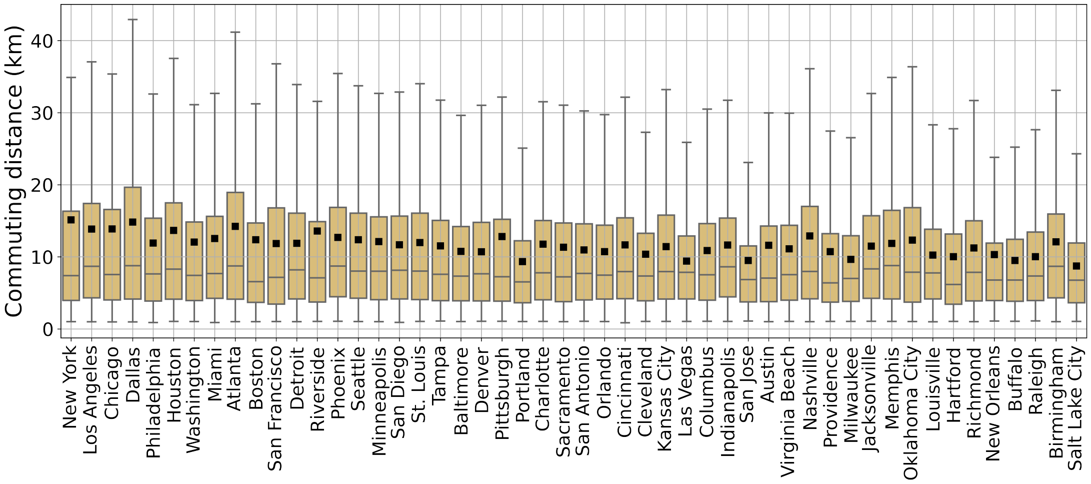


Figure 8: The distribution of commuting distances in the selected 50 metropolitan areas represented by boxplots. Black dots represent the average commuting distance in each metro area in our data. We only consider those users for whom home and work locations are identifiable, home and work is separated by a minimum 100 meter commute, and the user has minimum 1 friend with identified home and work locations in the same metro area. The metro areas are ordered by population and outlier individuals are not presented.

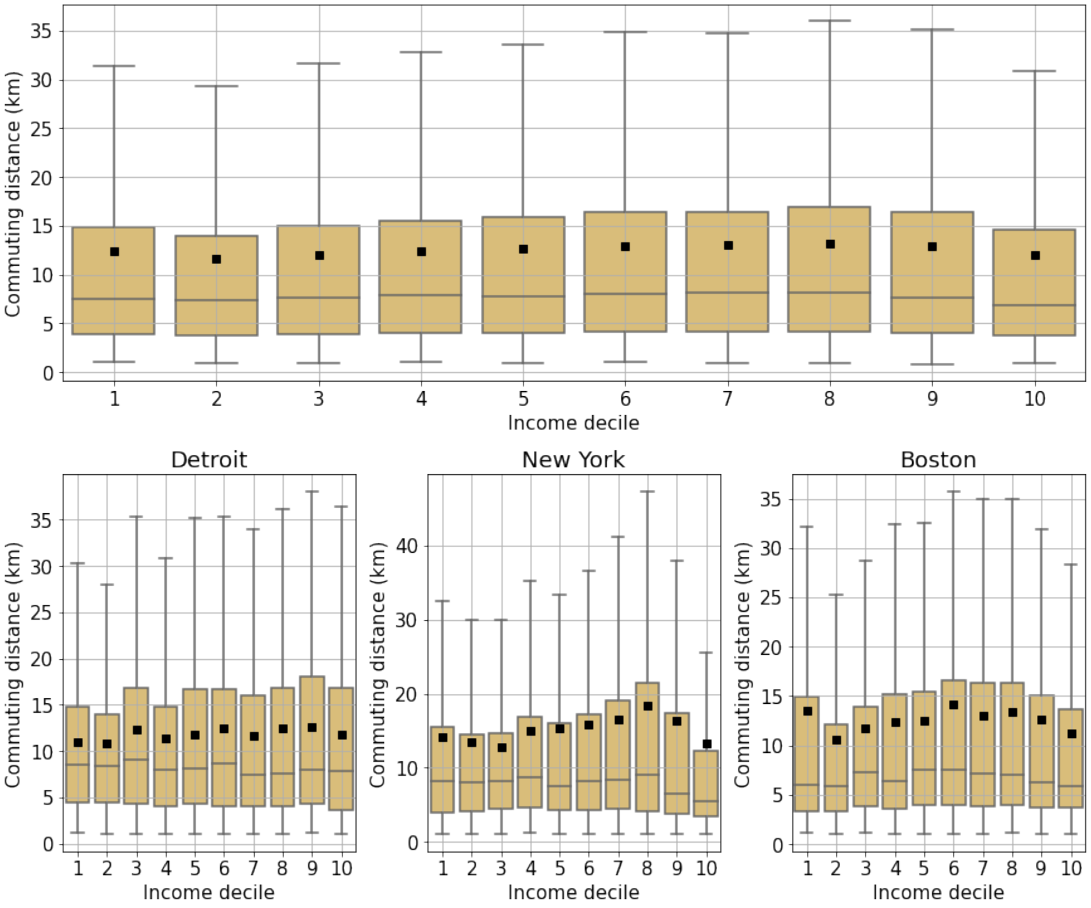


Figure 9: The distribution of commuting distances in different income deciles of cities across the selected 50 metropolitan areas represented by boxplots. Black dots represent the average commuting distance in each income decile. We only consider those users for whom home and work locations are identifiable, home and work is separated by a minimum 100 meter commute, and the user has minimum 1 friend with identified home and work locations in the same metro area. The first row plots the distribution of commuting grouped by income deciles and it show that the median and mean commuting distances are similar across income groups. The second row shows on the examples of Detroit, New York and Boston, that in particular cities different income groups commute slightly longer than others.

## SI 4: Effect of different distance thresholds

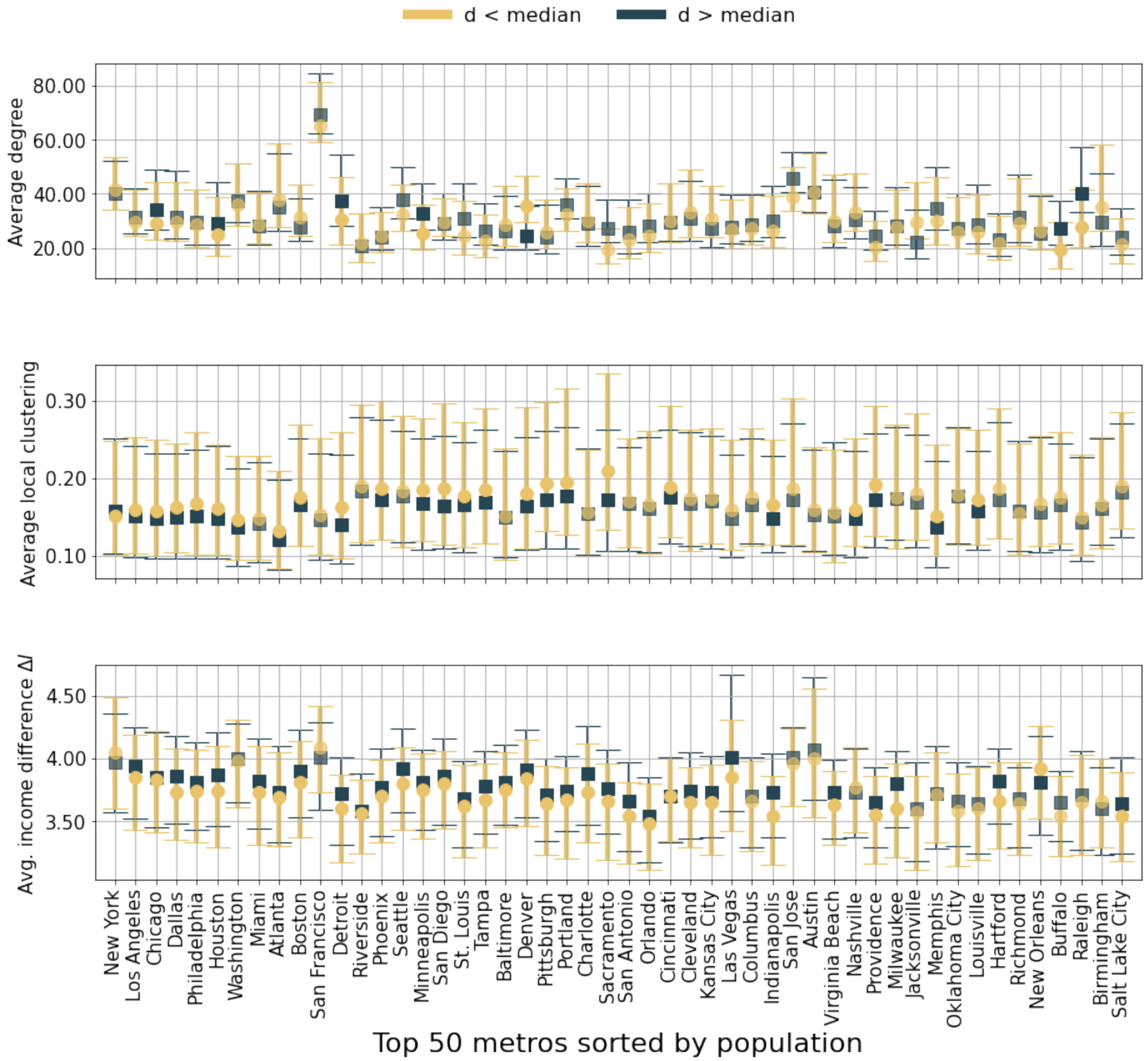


Figure 10: Network characteristics of users commuting above and below median distance in the top 50 metropolitan areas of the United States. The slightly more transparent signs indicate that t-tests suggest no significantly different means for the groups (p>0.05). Error bars represent the 25th and 75th percentile of the distribution for each group. To make the visualizations more appealing, we only plot group means in Figure 2 of the main text.

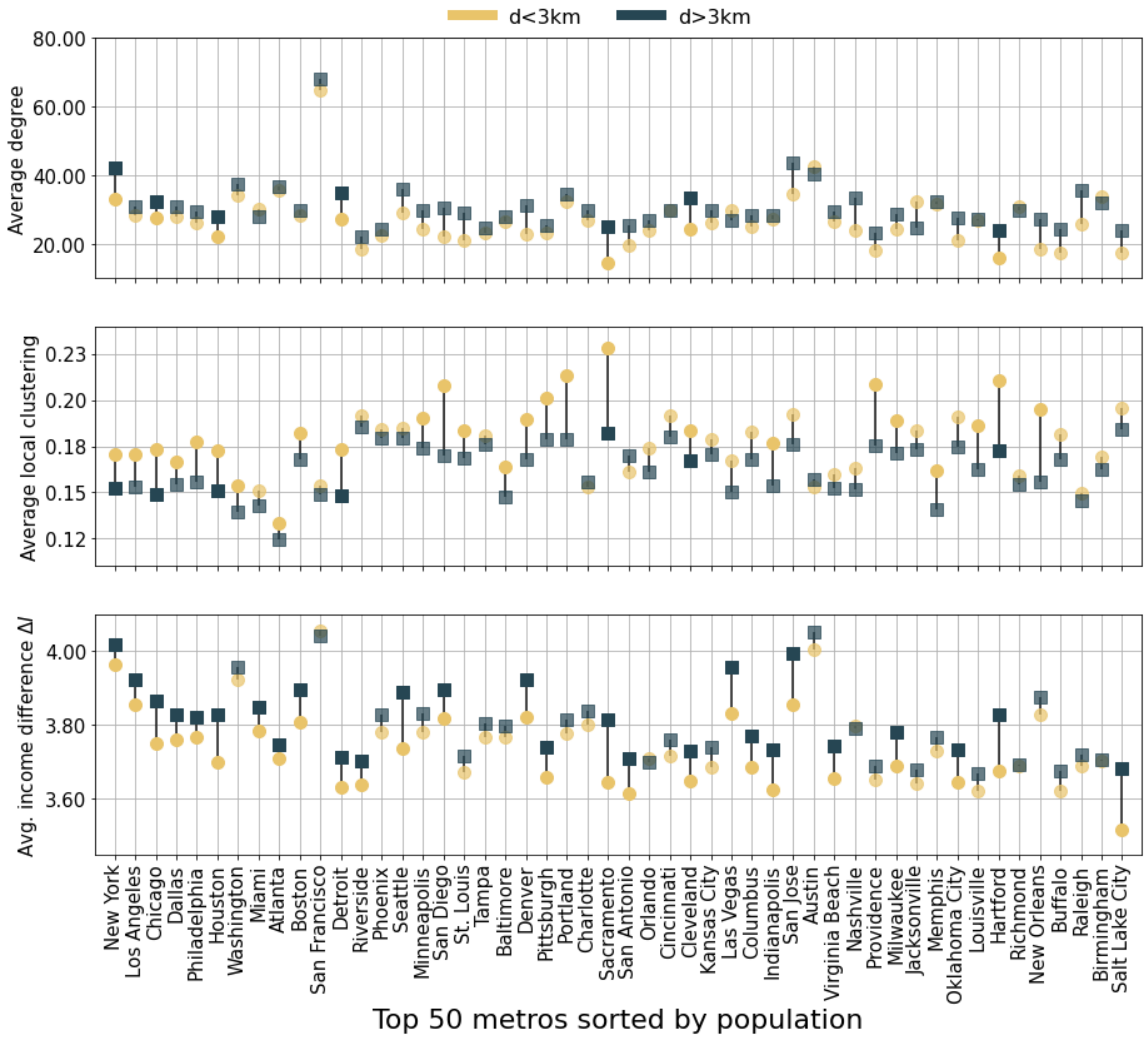


Figure 11: Network characteristics of users commuting above and below 3 km distance in the top 50 metropolitan areas of the United States. The slightly more transparent signs indicate that differences of means are not significant (p>0.05).

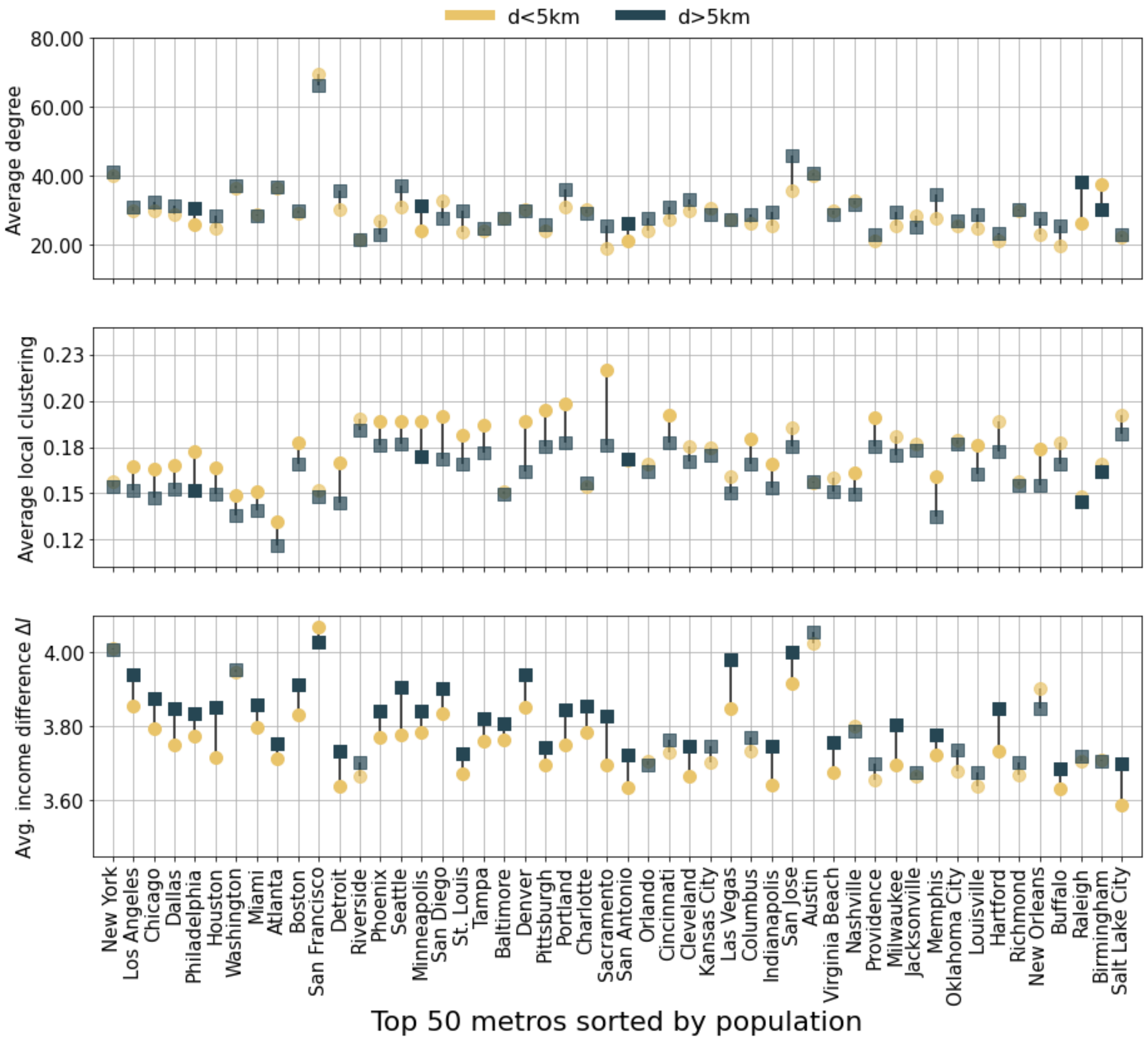


Figure 12: Network characteristics of users commuting above or below 5 km distance in the top 50 metropolitan areas of the United States. The slightly more transparent signs indicate that differences of means are not significant ($p>0.05$).

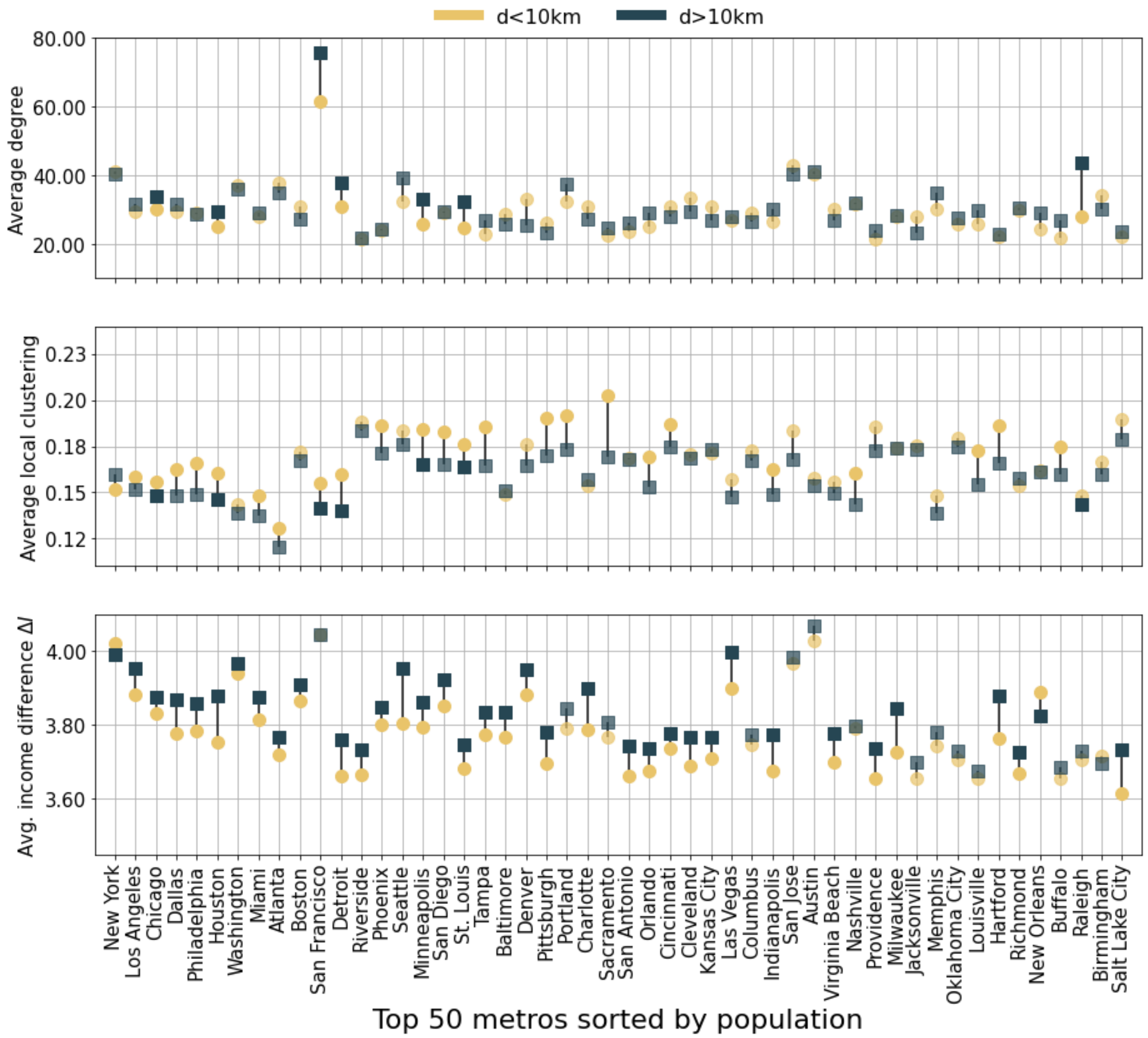


Figure 13: Network characteristics of users commuting above or below 10 km distance in the top 50 metropolitan areas of the United States. The slightly more transparent signs indicate that differences of means are not significant (p>0.05).

## SI 5: Regression on network characteristics and commuting

Table 1 presents 8 linear regression models to complement Figure 2 of the main text. For these robustness checks we log transferred (indicated in the table) or normalized the variables. We introduce control variables step-by-step. Model (1)-(4) further strengthens our previous findings at Figure 2a as longer commuting is connected to lower local clustering in the mutual followership ego network of users. However, the positive and significant quadratic term suggests that commuting distance has an increasing return on network clustering. This relationship is stable even while controlling for the degree, home income and metro area of users. Model (5) shows that longer commutes are linked to ego networks with lower income difference between friends and commuting distance has a diminishing return on income difference to friends. However, this relationship does not hold while controlling for the degree and home income of users in Model (8) whereas controllers introduced in Models (6)-(7) are stable.

Table 1: Relationship between commuting and network characteristics

| | *Dependent variable* | | | | | | | |
|---|---|---|---|---|---|---|---|---|
| | Local clustering | | | | Income diff. (log) | | | |
| | (1) | (2) | (3) | (4) | (5) | (6) | (7) | (8) |
| Distance (log) | −0.056*** | | | −0.036*** | 0.078** | | | −0.035 |
| | (0.003) | | | (0.003) | (0.031) | | | (0.028) |
| Distance$^2$ (log) | 0.023*** | | | 0.013*** | −0.059*** | | | 0.008 |
| | (0.002) | | | (0.001) | (0.016) | | | (0.014) |
| Degree (log) | | −0.178*** | | −0.179*** | | 0.368*** | | 0.422*** |
| | | (0.001) | | (0.001) | | (0.005) | | (0.005) |
| Income (log) | | | 0.018*** | −0.003** | | | 3.506*** | 3.546*** |
| | | | (0.001) | (0.001) | | | (0.013) | (0.013) |
| Constant | 0.155*** | 0.369*** | 0.049*** | 0.404*** | 1.049*** | 0.651*** | −14.394*** | −15.014*** |
| | (0.002) | (0.002) | (0.007) | (0.006) | (0.020) | (0.016) | (0.059) | (0.060) |
| Metro FE | Yes | Yes | Yes | Yes | Yes | Yes | Yes | Yes |
| Observations | 261,283 | 261,283 | 258,949 | 258,949 | 348,728 | 348,728 | 345,610 | 345,610 |
| $R^2$ | 0.009 | 0.243 | 0.007 | 0.244 | 0.009 | 0.023 | 0.182 | 0.200 |
| Adjusted $R^2$ | 0.008 | 0.243 | 0.007 | 0.244 | 0.009 | 0.023 | 0.182 | 0.200 |
| *Note:* | | | | | | | | *p<0.1; **p<0.05; ***p<0.01 |

Table 2 presents 3 additional linear regression models to uncover the relationship between the degree of users and their commuting distance. Results are in line with the trends of SI 4 Figure 8-10 as longer commuting is connected to higher degree, however, commuting distance has diminishing returns on user degree.

Table 2: Relationship between degree and commuting

| | *Dependent variable* | | |
|---|---|---|---|
| | Degree | | |
| | (1) | (2) | (3) |
| Distance (log) | 0.146*** | | 0.144*** |
| | (0.010) | | (0.010) |
| $Distance^2$ (log) | −0.074*** | | −0.073*** |
| | (0.005) | | (0.005) |
| Income (log) | | −0.096*** | −0.096*** |
| | | (0.005) | (0.005) |
| Constant | 1.047*** | 1.528*** | 1.474*** |
| | (0.007) | (0.021) | (0.021) |
| Metro FE | Yes | Yes | Yes |
| Observations | 348,728 | 345,610 | 345,610 |
| $R^2$ | 0.007 | 0.007 | 0.008 |
| Adjusted $R^2$ | 0.007 | 0.007 | 0.008 |
| *Note:* | | | *p<0.1; **p<0.05; ***p<0.01 |

## SI 6: All assortativity matrices for the top 50 US metropolitan areas

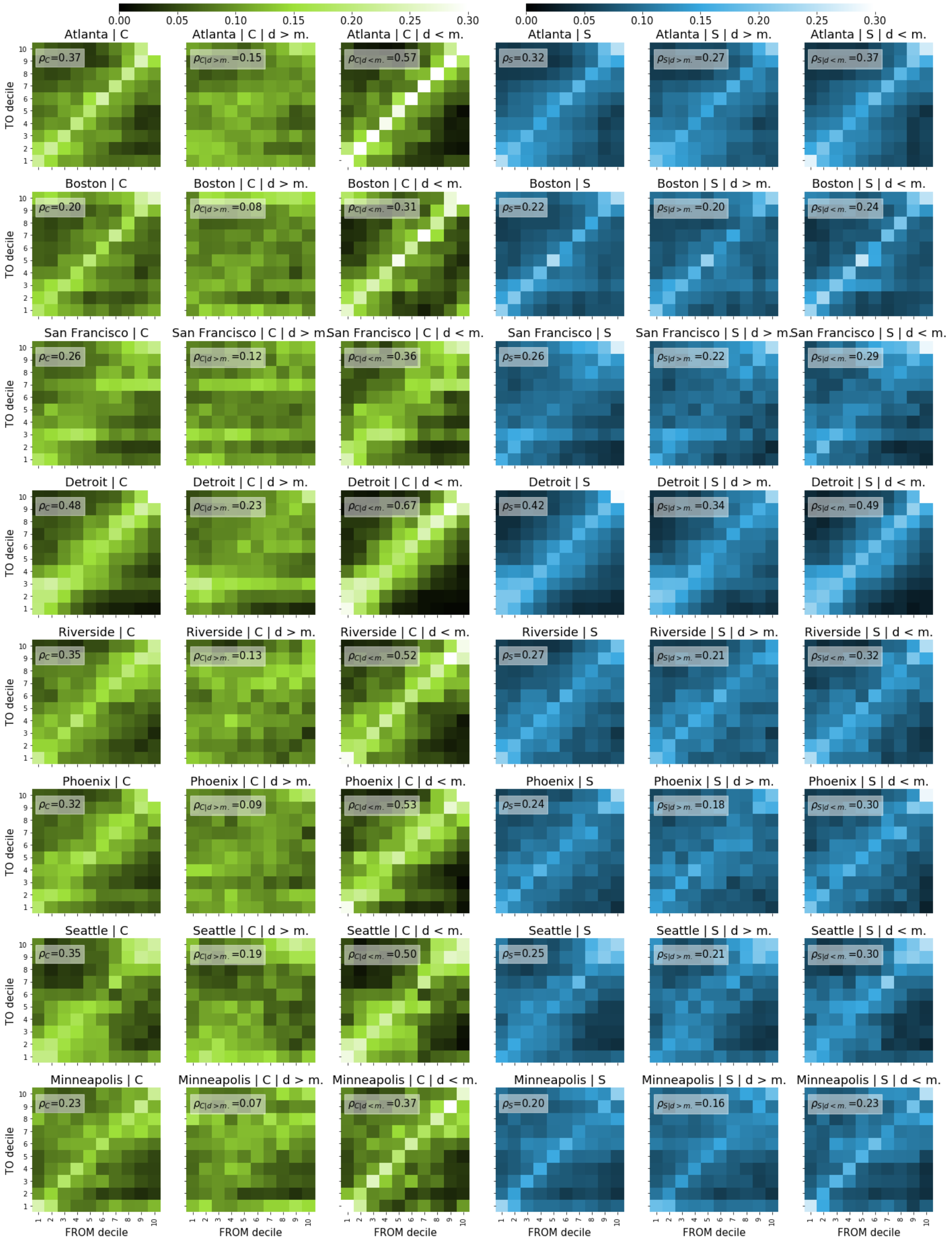


Figure 14: Assortativity matrices $C$ and $S$ in all 50 investigated US metropolitan areas for the overall, mobile and non-mobile users. $\rho$-values are indicated in the labels.

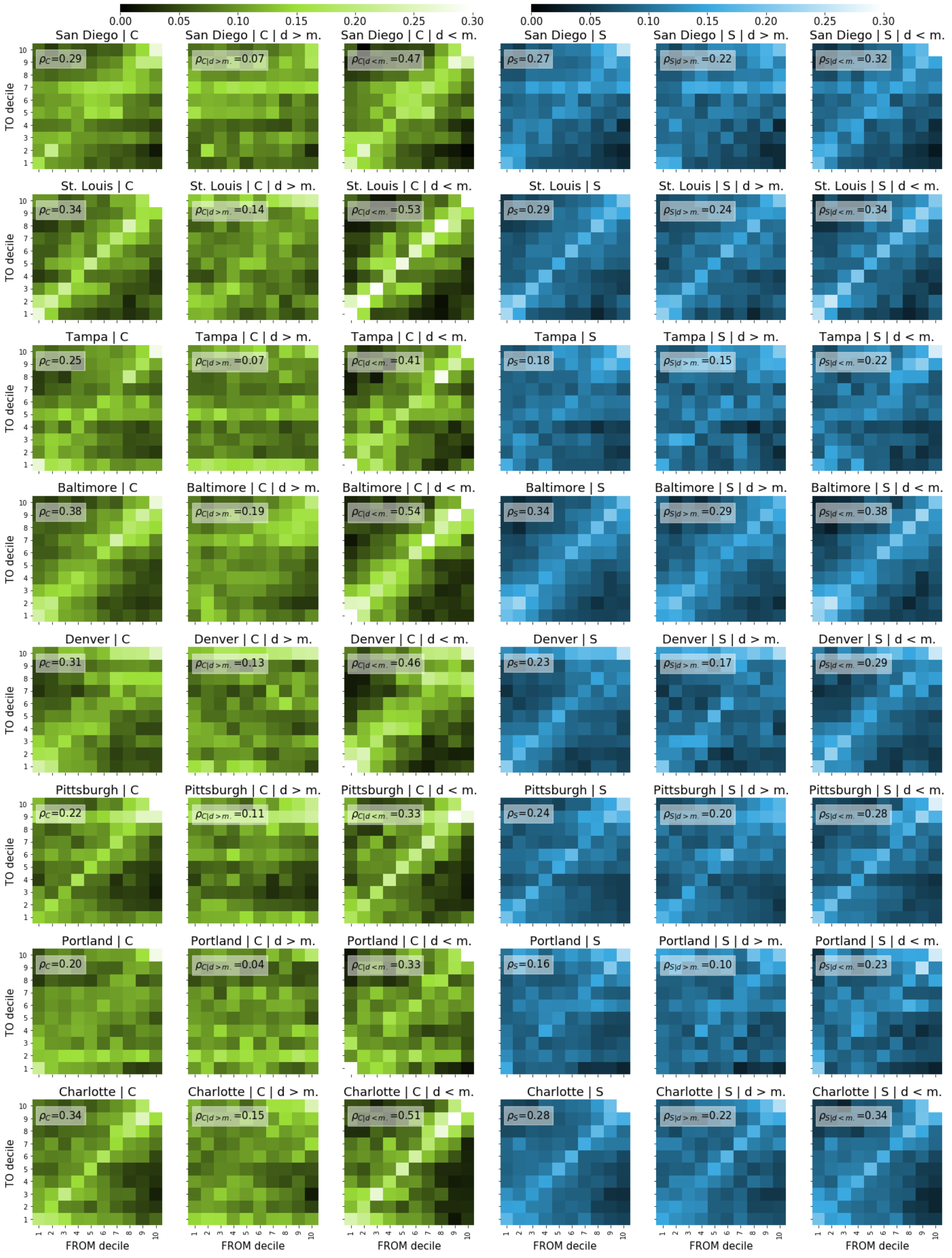


Figure 14: (continued) Assortativity matrices $C$ and $S$ in all 50 investigated US metropolitan areas for the overall, mobile and non-mobile users. $\rho$-values are indicated in the labels.

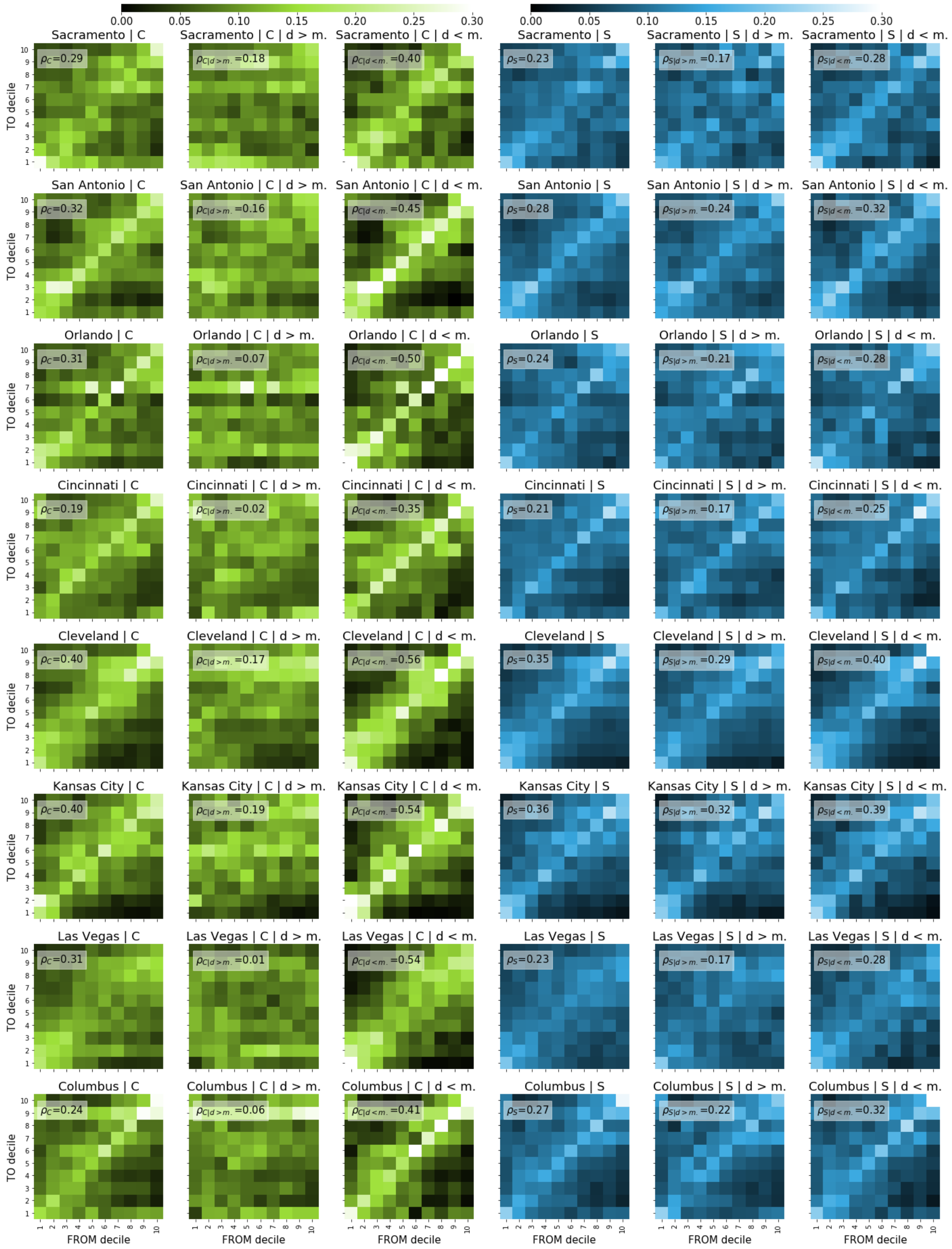


Figure 14: (continued) Assortativity matrices $C$ and $S$ in all 50 investigated US metropolitan areas for the overall, mobile and non-mobile users. $\rho$-values are indicated in the labels.

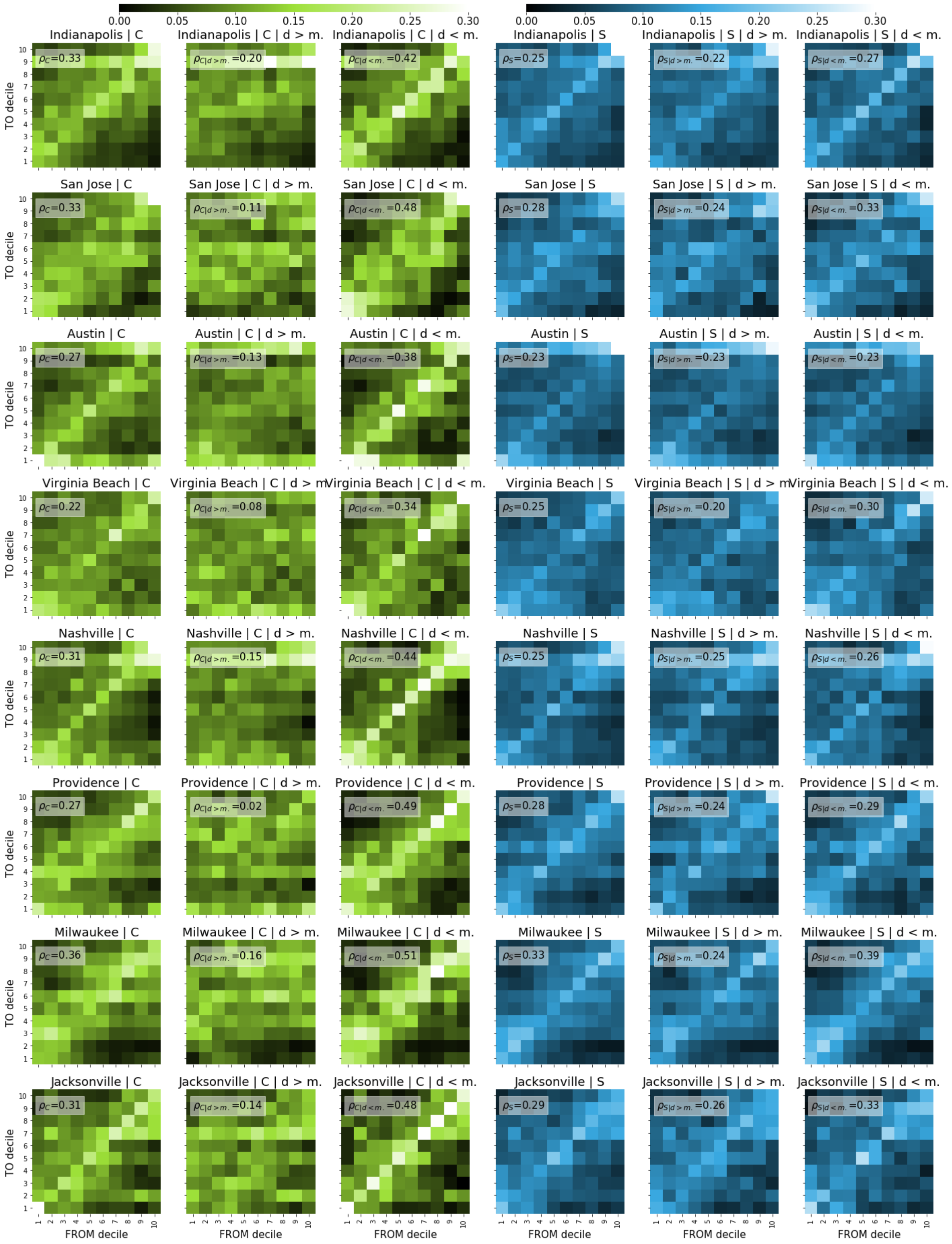


Figure 14: (continued) Assortativity matrices $C$ and $S$ in all 50 investigated US metropolitan areas for the overall, mobile and non-mobile users. $\rho$-values are indicated in the labels.

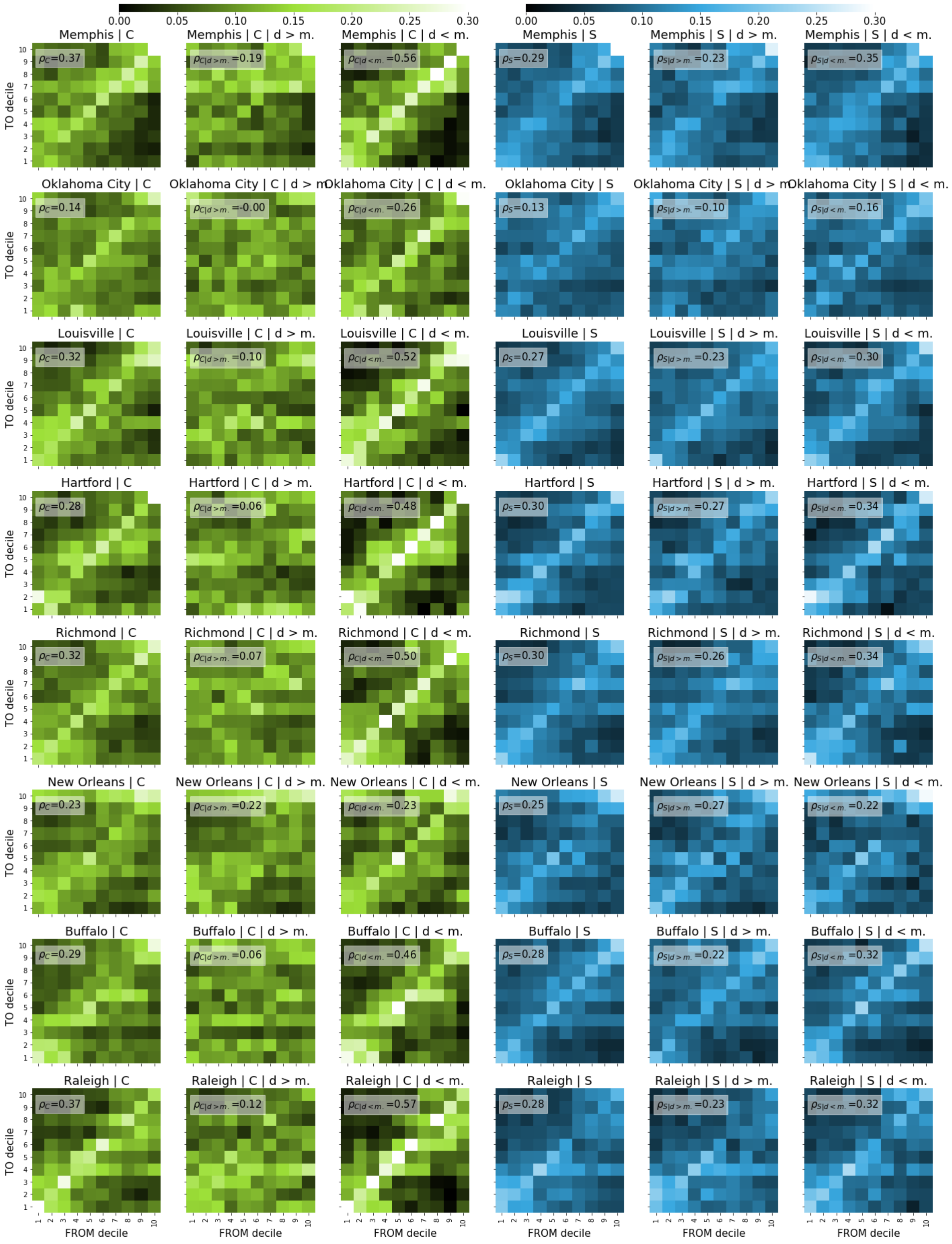


Figure 14: (continued) Assortativity matrices $C$ and $S$ in all 50 investigated US metropolitan areas for the overall, mobile and non-mobile users. $\rho$-values are indicated in the labels.

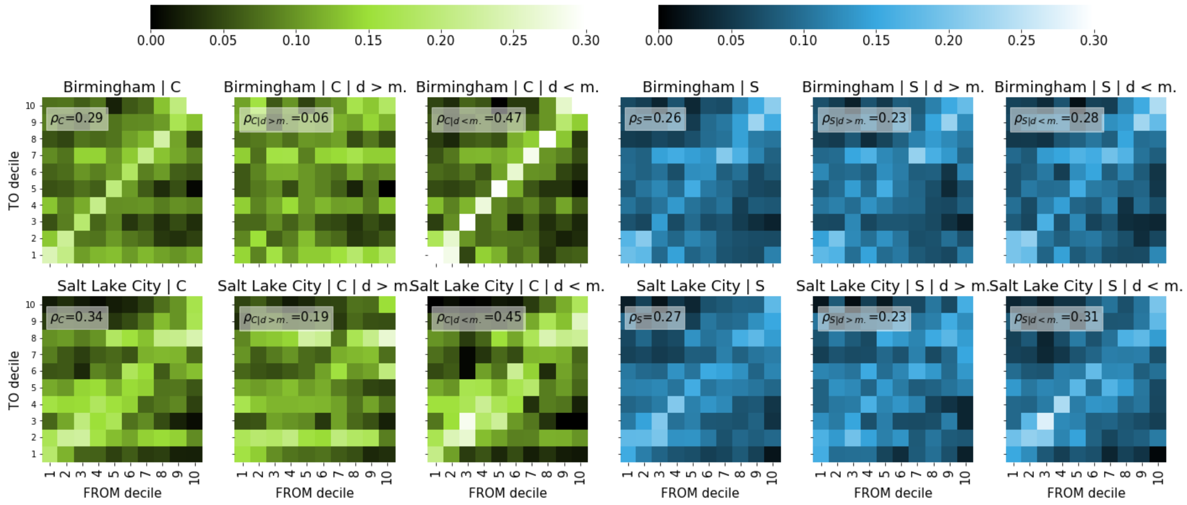


Figure 14: (continued) Assortativity matrices $C$ and $S$ in all 50 investigated US metropolitan areas for the overall, mobile and non-mobile users. $\rho$-values are indicated in the labels.

## SI 7: Different distance thresholds and assortativity change

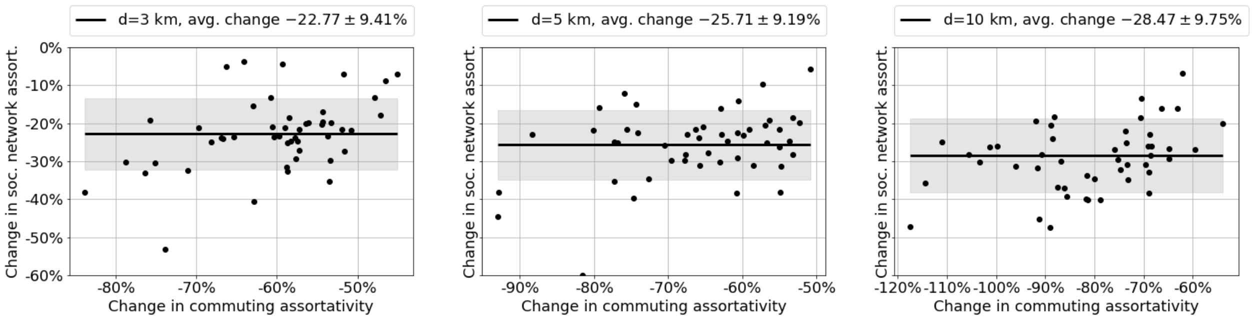


Figure 15: Change in the assortativity of the social network matrices vs. the commuting matrices for different distance thresholds

Figure 16 presents the average relative change in the assortativity values for the social network matrices, if instead of grouping users based on their commuting distance, we divide users into two randomly selected groups within cities in 50 realizations. The change is is on average 0.49% with a standard deviation of 1.57%. We did not control for any other variables in this randomization.

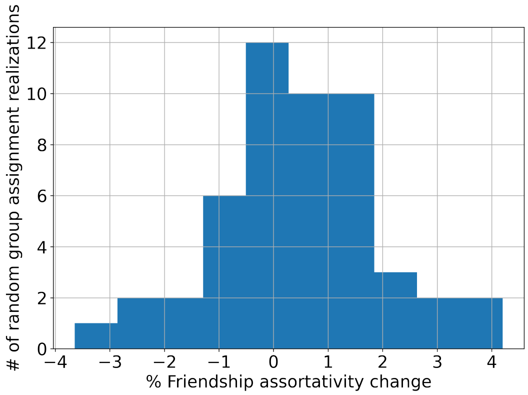


Figure 16: Average change in the assortativity of the social network matrices vs. the commuting matrices for two random user groups for 50 realizations

## SI 8: Diversity in commuting and social connections

We measure the diversity $S_C$ and $S_S$ of the matrices $C$ and $S$ (or for any matrices on the smaller user base, e.g. $S_{d>median}$) by averaging the normalized entropies of the columns of the matrices. Formally, for a $10 \times 10$ matrix $X$, where the sum of the columns $\sum_j X_{ij} = 1$ for every possible $j$,

$$S_X = \frac{1}{10} \cdot \sum_{j=1}^{10} \frac{1}{\log 10} \sum_{i=1}^{10} X_{ij} \cdot \log X_{ij}, \tag{1}$$

which means that $S_X = 0$ corresponds to a matrix in which every column contains exactly one element that is 1, and the others are 0, and $S_X = 1$ corresponds to the case when every element of the matrix is equal, $\frac{1}{10}$. Thus, $S_X$ values closer to 1 mean matrices in which commuting or friendship ties in a column are on average more distributed over multiple income classes, whereas smaller $S_X$ values mean matrix columns with rather one dominant element.

In parallel to the decreasing assortativity with longer commutes, we can observe an increasing average diversity for the connection patterns of both matrices, if measured by the averaged entropy of the column-wise probability distributions $S_S$ and $S_C$ (see Section **??** for details on this measure). This increase in the diversity is shown for all 50 metropolitan areas. Again, there is a higher increase in diversity for the commuting assortativity matrix, if we compare long commuters to short commuters, but this increase in the diversity is in parallel with the increase in the friendship assortativity matrix. If we measure which income deciled contribute to the increasing entropy values in both the mobility and the social network patterns, we can see that the lowest and highest income classes have the most diversity increase (see the inset). Therefore, it is most likely that rearrangement of the social connections of the richest and poorest deciles contribute most to the 30% decrease in social network assortativity that comes with longer commutes.

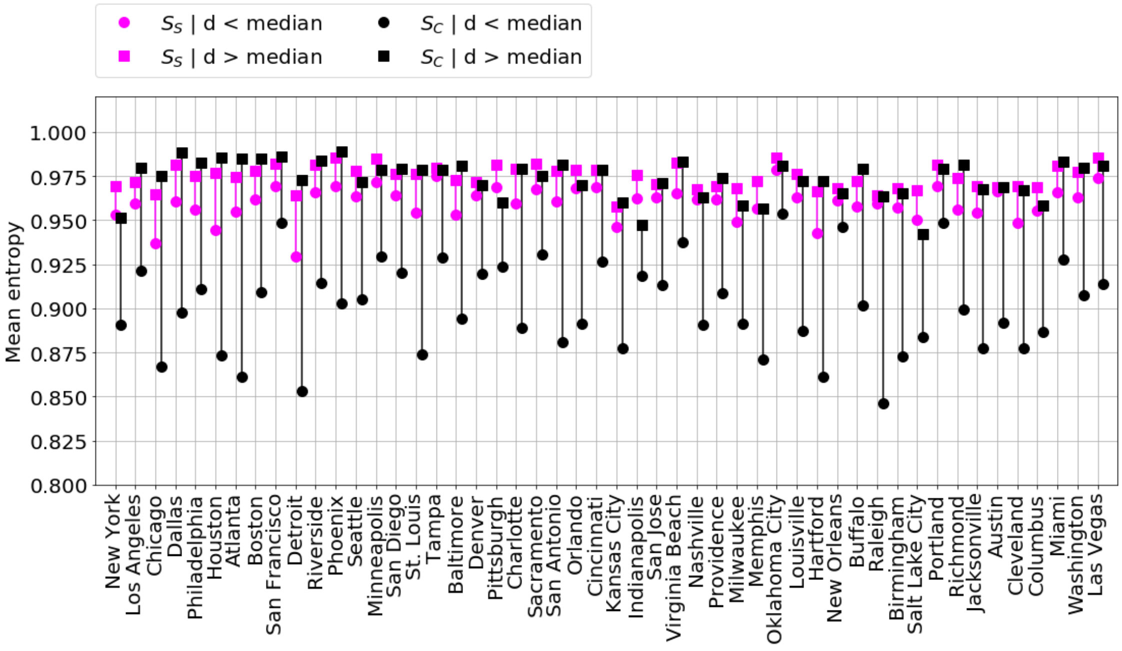


Figure 17: Diversity

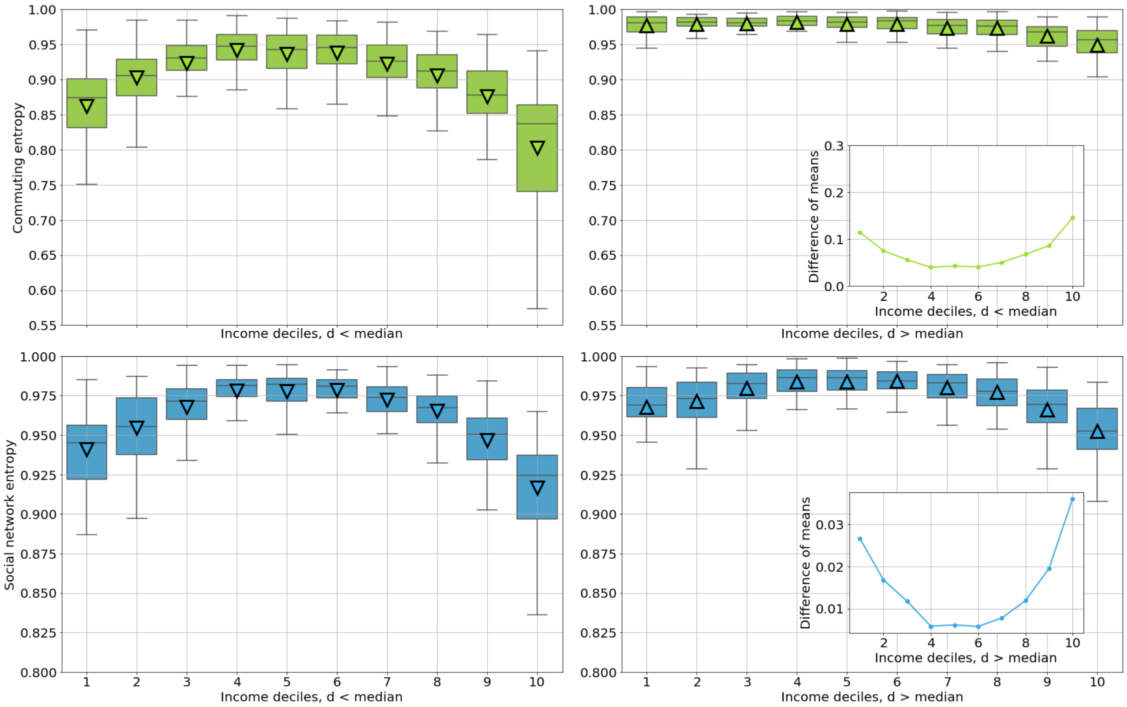


Figure 18: Assortativity of commuting and social network matrices by income groups.

## SI 9: Correlation in the commuting distances of social connections

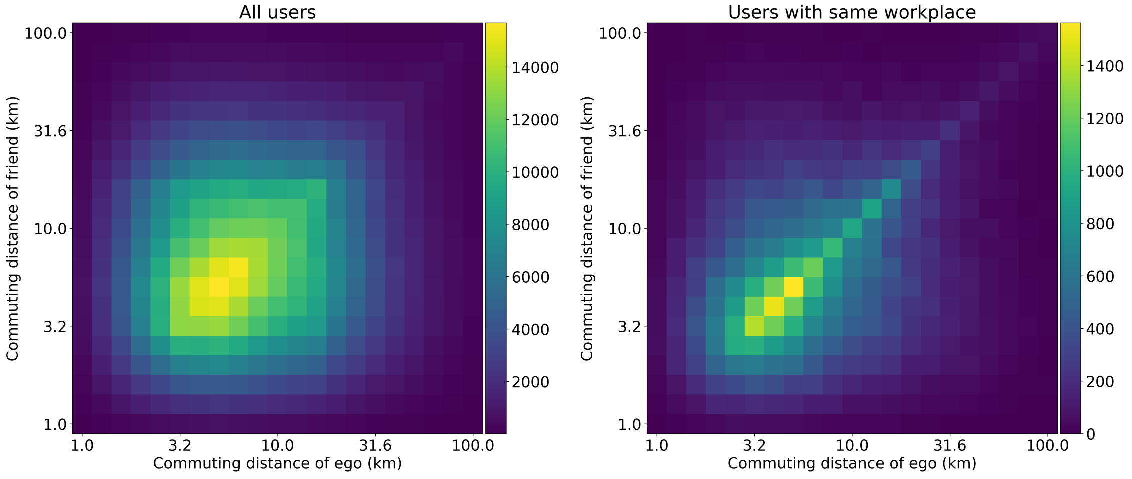


Figure 19: Illustration of correlation between mutual followers' commuting distances for all users (left) and for users having the same work census tract. Colorscale corresponds to number of user pairs falling into the given distance bins on the horizontal and vertical axes. Pearson correlation coefficients are $\rho = 0.11$ for all users, and $\rho = 0.34$ for users working in the same work census tract. This correlation illustrates that commuters tend to friend others who commute similar distances, especially if they work at identical locations. This is further sign of social assortativity that we demonstrate in the main text.

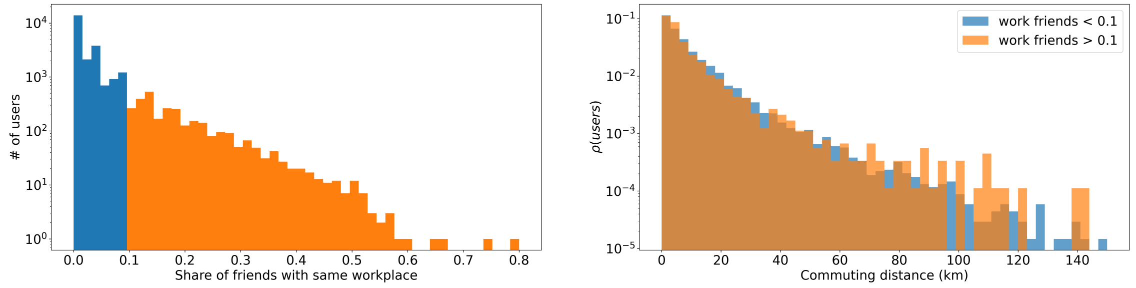


Figure 20: Left: the distribution of share of mutual followers having the same work census tract. Most user's have less than 10% of their mutual followers having the same work census tract (blue). Right: distribution of commuting distances for the $<$ 10% work friend (blue) and $>$ 10% work friend (orange) groups. Only the noisy tail differs, thus, commuting distances and share of work friends do not seem to be linked.

## SI 10: Excluding the effect of overlapping home and work census tract

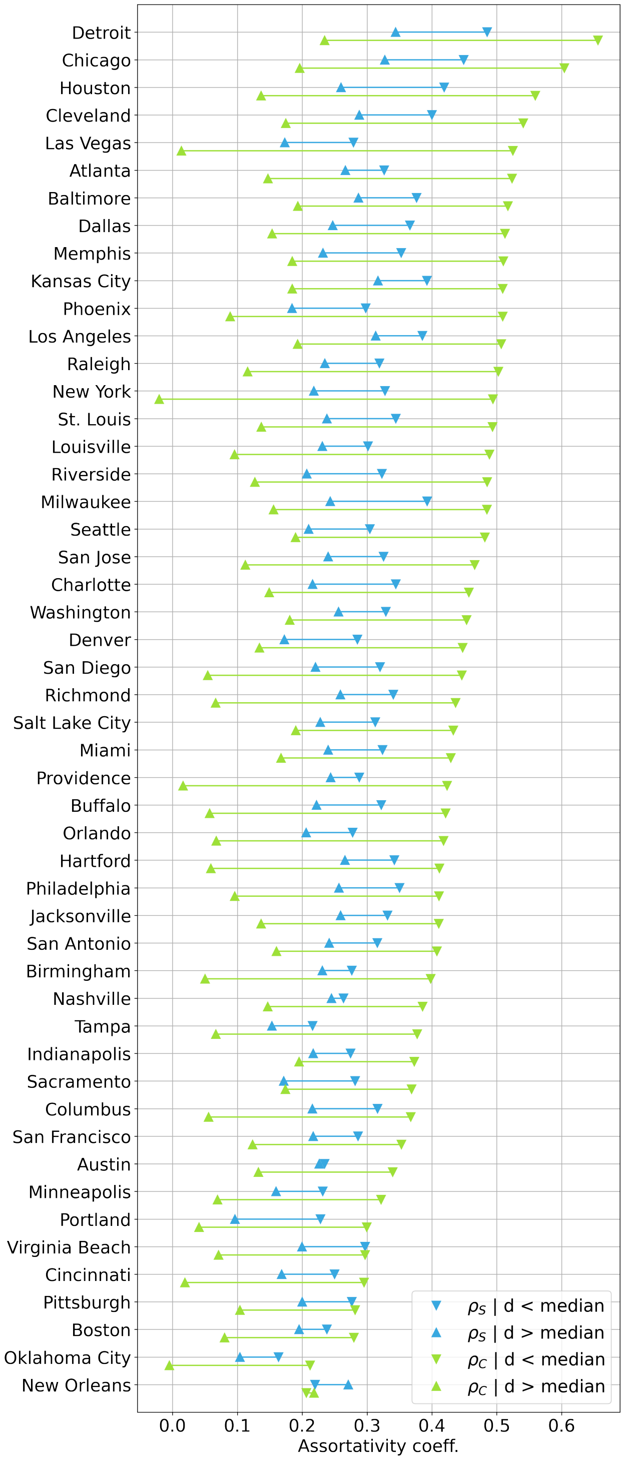


Figure 21: Reproduction of Figure 4g excluding users who commute more than 100 m, but their home census tract is the same as their work census tract.

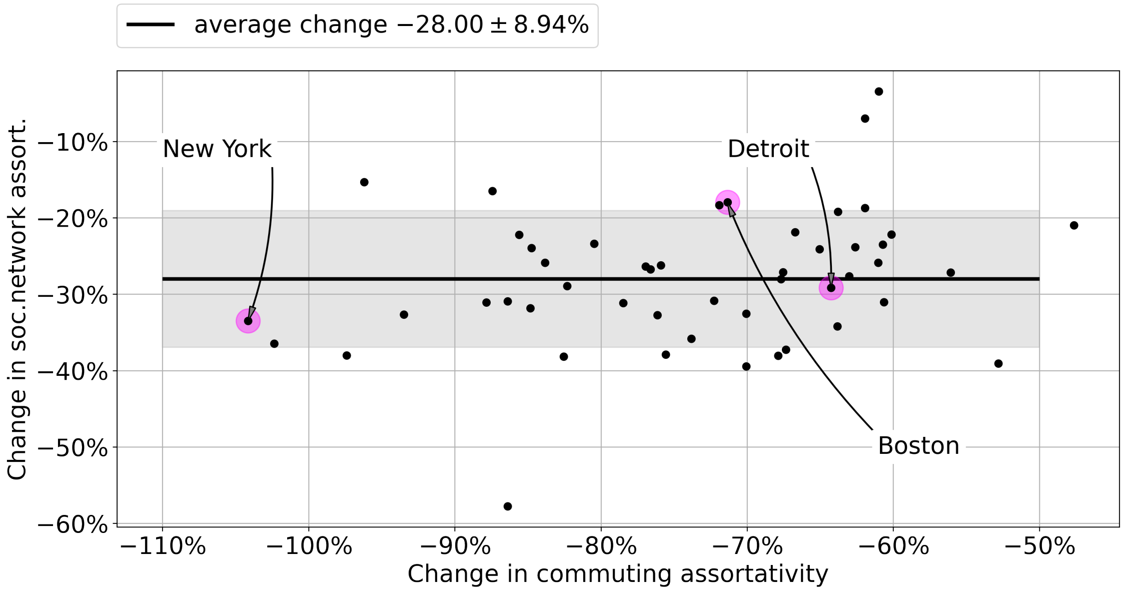


Figure 22: Reproduction of of Figure 4i excluding users who commute more than 100 m, but their home census tract is the same as their work census tract.

## SI 11: Home/work cluster detection errors

Home and work locations are estimated with the algorithm described in the Materials and methods section, that connects points that possibly belong to the same cluster if they are closer to each other than 1 km. After that, there's still a cluster searching and trimming phase, where outlier messages ($> 3\sigma$) are removed from clusters. The average distance of the geolocated messages measured from their corresponding cluster centers for 60 randomly selected clusters (mixed for home and work, as their detection only differs in the temporal domain, but not in the spatial) ranged from 4 m to 480 m, see the figure below, which is smaller than the average size of census tracts (in the top 50 investigated metro areas, $1.11 \pm 1.28$ km). Also, the majority of the cluster sizes was smaller than 100 m. Thus, the classification error of home and work locations into census tracts, and as such, income deciles is relatively low. Also, the size of these tweet clusters is negligible when compared to the overhead commuting distances (that again, fall behind actual distances), therefore, the accuracy of home and workplace estimation is unlikely to affect our results.

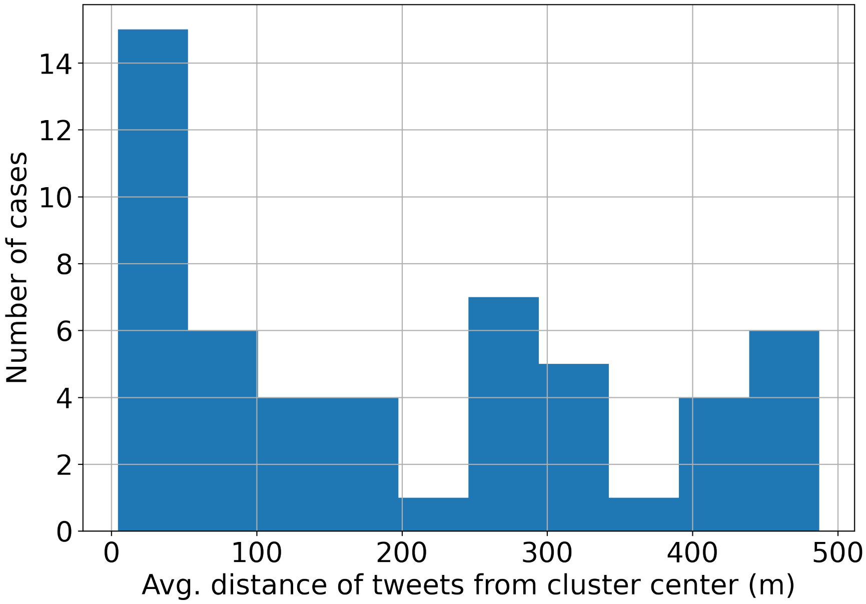


Figure 23: Average distances of geolocated messages from their corresponding cluster centers for 60 randomly selected clusters.

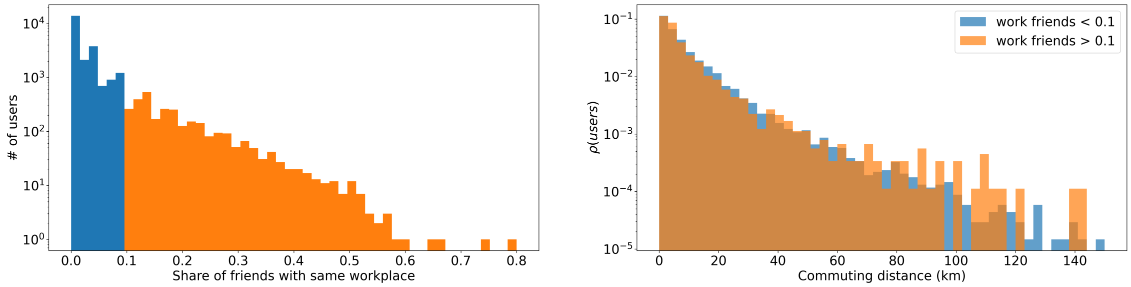


Figure 24: Left: the distribution of share of mutual followers having the same work census tract. Most user's have less than 10% of their mutual followers having the same work census tract (blue). Right: distribution of commuting distances for the $< 10\%$ work friend (blue) and $> 10\%$ work friend (orange) groups. Only the noisy tail differs, thus, commuting distances and share of work friends do not seem to be linked.
